# Similarities and differences in the gene expression signatures of physiological age versus future lifespan

**DOI:** 10.1111/acel.14428

**Published:** 2024-12-06

**Authors:** Matthew C. Mosley, Holly E. Kinser, Olivier M. F. Martin, Nicholas Stroustrup, Tim Schedl, Kerry Kornfeld, Zachary Pincus

**Affiliations:** ^1^ Department of Developmental Biology Washington University in St. Louis St. Louis Missouri USA; ^2^ Department of Biomedical Engineering Washington University in St. Louis St. Louis Missouri USA; ^3^ Centre for Genomic Regulation (CRG) The Barcelona Institute of Science and Technology Barcelona Spain; ^4^ Universitat Pompeu Fabra (UPF) Barcelona Spain; ^5^ Department of Genetics Washington University in St. Louis St. Louis Missouri USA; ^6^ Hexagon Bio Menlo Park California USA

**Keywords:** aging, longevity, systems biology, transcriptomics

## Abstract

Across all taxa of life, individuals within a species exhibit variable lifespans. Differences in genotype or environment are not sufficient to explain this variance, as even isogenic *Caenorhabditis elegans* nematodes reared under uniform conditions show significant variability in lifespan. To investigate this phenomenon, we used lifespan‐predictive biomarkers to isolate, at mid‐adulthood, prospectively long‐ and short‐lived individuals from an otherwise identical population. We selected two biomarkers which correlated positively with lifespan, *lin‐4*p::GFP and *mir‐243*p::GFP, and two which correlated negatively, *mir‐240/786*p::GFP and autofluorescence. The gene‐expression signature of long versus short future lifespan was strikingly similar across all four biomarkers tested. Since these biomarkers are expressed in different tissues, these results suggest a shared connection to a global health state correlated with future lifespan. To further investigate this underlying state, we compared the transcriptional signature of long versus short future lifespan to that of chronologically young versus old individuals. By comparison to a high‐resolution time series of the average aging transcriptome, we determined that subpopulations predicted to be long‐ or short‐lived by biomarker expression had significantly different transcriptional ages despite their shared chronological age. We found that this difference in apparent transcriptional age accounted for the majority of differentially expressed genes associated with future lifespan. Interestingly, we also identified several genes whose expression consistently separated samples by biomarker expression independent of apparent transcriptional age. These results suggest that the commonalities in the long‐lived versus short‐lived state reported across different biomarkers of aging extends beyond simply transcriptionally young versus transcriptionally old.

## Graphical abstract

The source of variability in lifespan among genetically identical *Caenorhabditis elegans* is a mystery. We used biomarkers of aging to isolate prospectively long‐lived and short‐lived individuals from the same population. We then used transcriptomic approaches to characterize these long‐ and short‐lived sub‐populations against the average physiological aging trajectory.

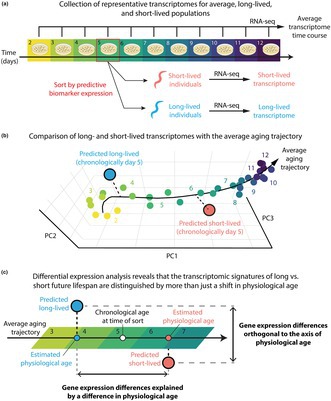

**Abbreviations**
C. elegans: Caenorhabditis elegans
GFP: Green fluorescent protein
PCA: Principal component analysis
RNA‐seq: RNA sequencing

## 1 INTRODUCTION

A fundamental and open question in the field of gerontology is the source of heterogeneity in lifespan among individuals of the same species. Even in model organisms, in which the variables of genetics and environment can be controlled, the mechanisms underlying the distribution of lifespans within a population remain a mystery. *Caenorhabditis elegans* is an excellent model species in which to study this phenomenon: they reproduce asexually as self‐fertile hermaphrodites, facilitating the generation of genetically homogenous populations, their lifespans are short, and they are reared in a (relatively) simple and easy‐to‐control lab environment. In spite of this genetic and environmental uniformity, however, variability in lifespans among *C. elegans* is comparable to that observed in genetically diverse humans (Kirkwood et al., 2005; Vaupel et al., 1998; Zhang et al., 2016). If not the twin phenotypic pillars of genetics and environment, what can account for this variability? One theory is that stochastic differences such as random damage or small fluctuations in concentration of key regulatory proteins set individuals on different paths as early as development (Kirkwood et al., 2005). It has even been argued that susceptibility to such fluctuations could in fact be adaptive as a form of bet‐hedging: variability in gene expression across a population, leading to a distribution of different phenotypes among a genetically identical population, could increase the likelihood of survival in the face of unknown future environments, with variability in lifespan as a (likely un‐selected‐for) side effect (Martin, 2009). Another (not mutually exclusive) theory is that organismal homeostasis is maintained by a web of interconnected systems, and individuals are canalized into one of a discrete number of failure states depending on which critical nodes are the first to fail (Cohen et al., 2022). In any case, investigating the ways in which different individuals age is a necessary step toward understanding the basis of this heterogeneity.

The aging process in *C. elegans* has been studied in great detail—its transparent body and microscopic size has facilitated phenotypic characterization at the cellular, physiological, and behavioral levels. Consequently, several biomarkers of aging have been determined for *C. elegans*: phenotypic measurements which vary with an individual's rate of aging and thus can be used to predict that individual's future lifespan (Baker & Sprott, 1988; Son et al., 2019). Many biomarkers can be intuitively understood as a measure of functional capacity or physiological robustness. For instance, movement (Golden et al., 2008; Herndon et al., 2002; Martineau et al., 2020; Zhang et al., 2016), pharynx pumping (Johnston et al., 2008), and mated progeny production (Pickett et al., 2013) all decline with age across the population but decline more rapidly in shorter‐lived individuals.

Even in an initially synchronized population, as each individual proceeds along a trajectory from robustness to frailty at different rates, a distribution of health states will emerge among individuals of the same chronological age. In contrast to chronological age, an individual's position along this robust‐to‐frail axis can be considered as its physiological age. Biomarkers of aging provide metrics by which this state can be measured relative to the rest of the population. Such biomarkers are thus powerful tools in the effort to understand interindividual heterogeneity in the aging process because they allow us to probe the underlying biology that might lead some animals to retain a youthful physiology while others in the same cohort experience premature decline and, ultimately, an earlier demise.

In addition to the tissue‐level and behavioral biomarkers mentioned above, many molecular biomarkers of aging have been identified as well. For instance, several measures of stress response such as induction of heat shock response (Rea et al., 2005), response to bacterial pathogenesis (Sánchez‐Blanco & Kim, 2011), and early‐life oxidation (Bazopoulou et al., 2019) have been shown to correlate with future lifespan. More recently, in a survey of *C. elegans* strains carrying GFP reporters driven by various microRNA promoters, Kinser et al. identified several whose expression in midlife correlates with lifespan at the individual level (Kinser et al., 2021). At least three of these reporters predicted lifespan in a redundant manner—that is, the information gained about future lifespan by measurement of one reporter was not enhanced by measurement of another, suggesting a shared connection to the same underlying pathway(s) or process(es) that act to determine lifespan. This finding was surprising given that the reporters were expressed in distinct tissues, implying some cell‐nonautonomous communication. Furthermore, none of the reporters which were tested in a mutant for *daf‐16*, a key regulator of longevity in *C. elegans*, required its activity in order to predict lifespan. This result implies that the relationship between reporter expression and lifespan was not dependent on differential expression of *daf‐16* and, by extension, differential activity of the insulin/insulin‐like signaling pathway. Taken together, these results suggest a potentially cell‐nonautonomous, organism‐wide process which governs the rate of aging, or at least a global health state to which each predictive biomarker was linked.

Given the apparent redundancy in the ability to predict lifespan in biomarkers tested by Kinser et al., we hypothesized that the global gene expression landscape underlying the expression of these different biomarkers of aging would be similar to one another. Furthermore, if the biomarkers in question are indeed linked to some common underlying program, we hypothesized that such a state would correlate with a difference in physiological age, measured via gene expression—that is, predicted‐short‐lived individuals would have gene‐expression patterns similar to older populations, while predicted‐long‐lived individuals would be similar to younger populations.

To test this, we measured a time‐series of RNA‐sequencing data from large synchronized populations at days 2–12 of life to define an average “reference” expression pattern for individuals of a given chronological age. Using this reference, we define the physiological age associated with any “query” gene‐expression measurement (regardless of the chronological age of the individuals from which the RNA was extracted) as the age of the reference population that most closely matches the query. We then sorted synchronized populations at Day 5 into predicted long‐ and short‐lived subpopulations using four biomarkers of aging: *lin‐4*p::GFP, *mir‐243*p::GFP, *mir‐240/786*p::GFP, and gut autofluorescence. After sorting, we performed RNA‐seq on these subpopulations (Figure S1a).

Overall, we found that the signatures of long versus short future lifespan predicted by each biomarker tested were indeed similar to one another. By “signature,” we mean the characteristic pattern of mRNA levels observed by parallel measurement of several thousand mRNA transcripts. This is consistent with previous data from Kinser et al. (2021) and supports the conclusion that, despite their expression in different tissues and opposite correlations with future lifespan, these biomarkers are connected through a common underlying transcriptomic landscape. Moreover, we found that this common signature is largely reflective of a shift along the axis of physiological age, suggesting that, at the gene expression level, short‐lived animals are indeed aging faster than long‐lived animals (Figure S1b). Unexpectedly, however, in comparing the predicted long‐ versus short‐lived subpopulations to the average transcriptomic aging trajectory, we also found a subset of differentially expressed genes which distinguished samples by biomarker expression in a manner orthogonal to physiological age (Figure S1c). Like the shared correlation with physiological age, this second class of genes was also shared across all of the biomarkers tested. This suggests, first, that these are not simply the chance correlations with biomarker status that would be expected in any dataset of a similar size, and second, that the commonalities between cohorts defined by these different biomarkers are due to more than just a shared shift in physiological age. These results lend credence to the idea that long‐ and short‐lived individuals of the same genetic background show distinct patterns of gene expression, which may in turn cause them to proceed at different rates along a common aging trajectory.

## 2 RESULTS

### 2.1 Construction of the average population aging transcriptome

To test the hypothesis that biomarker expression in longer‐ versus shorter‐lived subpopulations correlates with an underlying state of physiological age, we first characterized the aging process in the average population. We collected and sequenced RNA from large populations aged days 2–12, counting first exposure to food after synchronization by hypochlorite treatment as Day 0. Cultures were maintained at 25°C, and all strains carried a temperature sensitive *spe‐9* mutation, which prevents fertilization but does not otherwise affect spermatogenesis, oogenesis, or lifespan (Fabian & Johnson, 1994; Singson et al., 1998). We know from measurements of biomarkers of aging that heterogeneity exists within the population such that, at each timepoint, we are sampling individuals with a range of health states. However, because the whole population is pooled, the RNA‐seq profile at each timepoint corresponds to the overall average transcriptional state of individuals at that chronological age.

**FIGURE 1:**
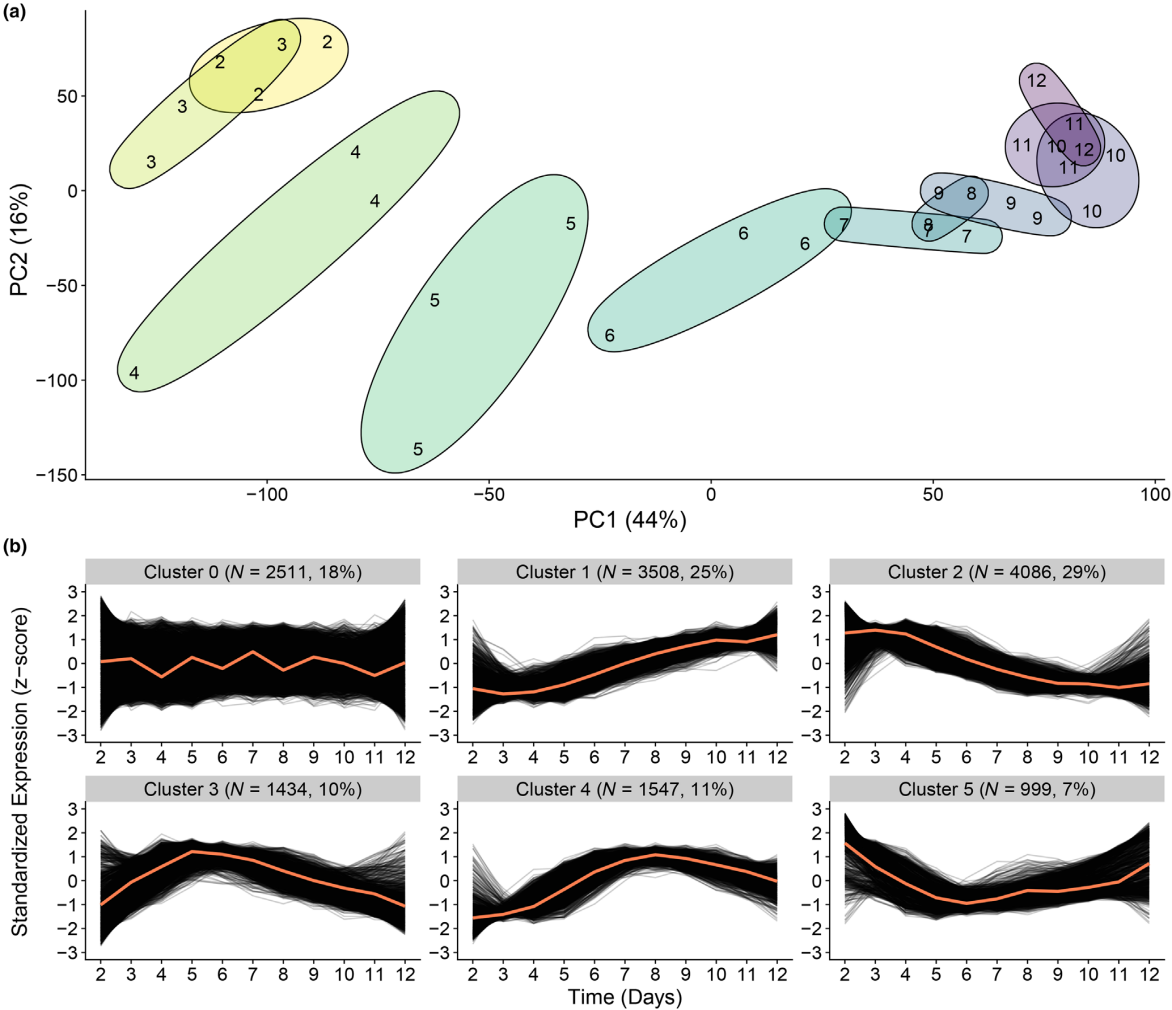
Trajectory of aging in the average population. (a) PCA plot of RNA‐seq data for populations at days 2–12 of life. Axis labels indicate percent of variance explained. Ellipses are drawn to enclose datapoints of the same chronological age. (b) Hierarchical clustering of temporal gene expression trajectories. Expression is plotted in terms of *z*‐score such that 0 is the mean and units represent standard deviations from the mean for each gene. Cluster centroids are plotted in orange. Plots are labeled with the number of genes that fall into each cluster and what percent of all detected genes that number represents. “Cluster 0” represents all genes whose expression did not correlate significantly with time.

We performed principal component analysis (PCA) to visualize the trajectory of gene expression over time. We observed that the first principal component (PC) generally distributed samples by age, while the second and third PCs describe a roughly U‐shaped trajectory through time (Figure 1a and Figure S2). To further characterize the average aging process, we performed hierarchical clustering to group together genes with similar temporal dynamics.

For each gene, expression levels over time were standardized to have the same mean and standard deviation, allowing for comparison even between genes which are expressed at vastly different levels. Thus, we are comparing genes which undergo similar proportional, rather than absolute, changes. We grouped genes into six clusters (Figure 1b), with cluster 0 defined as genes whose expression did not show significant correlation with time. Over half of all detected genes were classified into clusters 1 and 2, describing roughly monotonic trends upwards or downwards over time, respectively. Enrichment analysis with WormCat (Holdorf et al., 2020) showed that cluster 1 was associated the cytoskeletal genes, signaling molecules, and genetic regulators, while cluster 2 appeared to largely represent a decline in metabolic and proteostasis genes (Figure S3b,c). Cluster 3 contained genes whose expression peaked by days 4–5 and were primarily associated with stress response (Figure S3d), while genes in cluster 4 peaked later in life and were enriched primarily for transcription factors and signaling molecules (Figure S3e). Cluster 5 described the opposite, with genes whose expressions reach their minimum in mid‐life before rising slowly over time, and was enriched primarily for ribosome biogenesis genes (Figure S3f).

### 2.2 Isolation of long‐ and short‐lived subpopulations by biomarker expression

We employed fluorescent biomarkers of aging to separate prospectively long‐ or short‐lived individuals from the rest of the population for two reasons. First, and pragmatically: in comparison to phenotypes such as movement or pharynx pumping, fluorescent markers are very quick to measure, which facilitates the rapid sorting of large populations. Second, and on more conceptual grounds: we expect differences in GFP expression levels of these biomarkers among individuals to necessarily reflect differences in transcriptional state. (If the differences in GFP levels were due to post‐transcriptional influences on GFP translation, folding, degradation, or other property, then all GFP transgenes expressed in the same cells/tissues as the biomarkers identified by Kinser et al. (2021) would share the same lifespan‐predictive properties—which is not the case). Therefore, we expect that there should be a distinct transcriptional signature underlying high versus low GFP fluorescence.

In order to select biomarkers of aging with which to separate long‐ and short‐lived subpopulations, we reanalyzed data from Kinser et al. (2021), which assessed a variety of transgenic microRNA promoter::GFP fusion constructs for their ability to predict future lifespan. Animals were reared individually and imaged longitudinally throughout their lives (Pittman et al., 2017). Because they had access to longitudinal measurement, Kinser et al. were able to use measurements at multiple timepoints (e.g., an individual's rate of change in fluorescence intensity) to show correlation with lifespan. However, in order to sort a large population reared together, we required biomarkers whose fluorescence would be sufficiently predictive of lifespan to separate long‐ versus short‐lived individuals with a single measurement. To identify such markers, we performed regression analysis at each timepoint to measure the correlation between fluorescence intensity and eventual lifespan (Figure S4).

We chose four biomarkers whose fluorescence in mid‐life correlated significantly with future lifespan. Three were integrated fluorescent reporters of gene expression, specifically the promoter sequence of the microRNAs *lin‐4*, *mir‐243*, and *mir‐240/786* driving GFP expression (Figure S4a–c). The fourth was not a genetic marker but rather naturally‐occurring autofluorescent materials which accumulate over time, specifically imaged in the red channel using a TRITC filterset (henceforth simply called “autofluorescence;” Figure S4d) (Pincus et al., 2016). Of these, *lin‐4*p::GFP and *mir‐243*p::GFP fluorescence correlate positively with lifespan, while *mir‐240/786*p::GFP fluorescence and autofluorescence correlate negatively with lifespan. Further, all of these biomarkers best predict lifespan between ~5–8 days post hatch, when more than 90% of the population is still alive (Figure S4, right column). *lin‐4*p::GFP is expressed primarily in the posterior intestine, *mir‐243*p::GFP is ubiquitous throughout the intestine, autofluorescence can be seen in the intestine and uterus, and *mir‐240/786*p::GFP expression is limited to the spermatheca (Figure S5).

**FIGURE 2:**
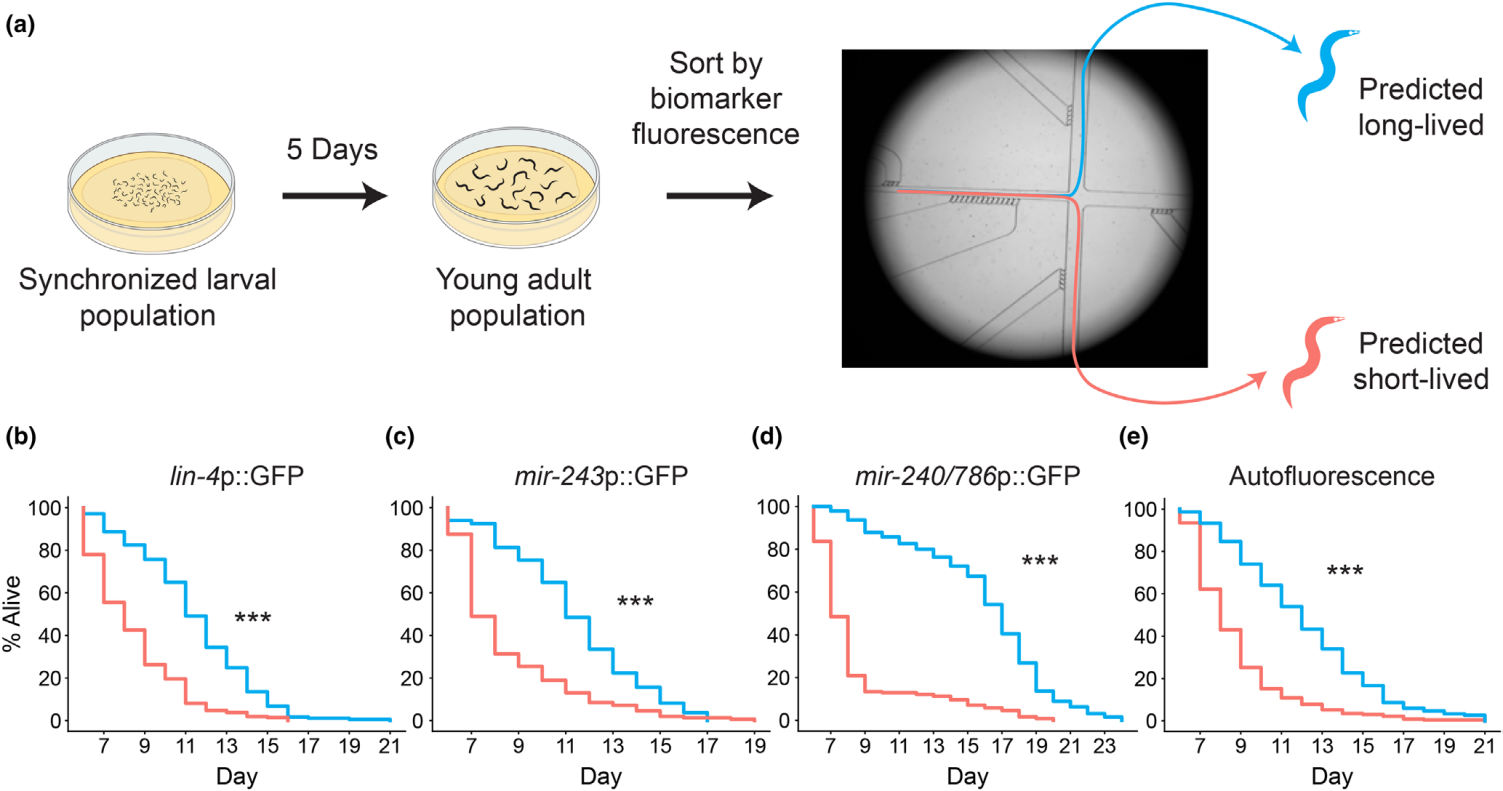
Separation of long‐lived and short‐lived animals from the average population. (a) Synchronized isogenic populations were cultured at 25°C for 5 days at which point they were collected for sorting. Individuals were immobilized and imaged on a microfluidic chip, and fluorescence was deemed “high” or “low” if an individual fell within the top or bottom tenth percentile, respectively, of fluorescence intensity relative to the rest of the population. (b–e) Lifespan curves comparing post‐sorting survival of predicted long‐lived (blue) and predicted short‐lived (red) sub‐populations of four biomarkers. In each case, the difference in survival was determined to be statistically significant by log‐rank test (*** = *p* < 0.0001). Curves are plotted starting at Day 6 (1 day‐post sorting), and each curve represents two combined replicates. Summary statistics for these experiments can be found in Table S1.

We sorted animals by fluorescence of each of these four biomarkers. Populations of age‐synchronized animals expressing each marker (or none in the case of autofluorescence) were allowed to grow for 5 days, at which point they were sorted using a single‐layer microfluidic system developed in our lab (Figure 2a and Figure S6). During the sorting process, each animal was immobilized, imaged, and routed down one of three output channels based on fluorescence intensity relative to the rest of the population. “High” and “low” fluorescence populations, defined as animals in the top or bottom tenth percentile of fluorescence intensity, respectively, were retained for further analysis. The remaining population, constituting the middle 80% of fluorescence was discarded.

We carried out survival assays on the sorted high‐ and low‐fluorescence subpopulations to confirm that this single‐timepoint measurement of biomarker fluorescence was sufficient to differentiate animals by future lifespan, and that the markers remained predictive outside the context of single‐animal culture. In each case, we found significant differences in lifespan associated with biomarker level, ranging from 20% to 137% differences in median lifespan between predicted long‐lived and short‐lived groups (Figure 2b–e and Table S1). While we were able to enrich for long‐ and short‐lived worms in this way, we still noted considerable variability even among these sorted groups.

We performed RNA‐seq on additional subpopulations sorted in the same manner. We used PCA to visualize the differences between samples and sample groups (Figure S7). The first principal component explained 44% of the variance and clearly separated sample groups by their predicted lifespans. Subsequent PCs, meanwhile, each explained less than 7% of the variance. Sample groups did not appear to distribute by biomarker type but only by biomarker level. From this we concluded that subpopulations with similar predicted lifespans are broadly similar to one another at the transcriptomic level, irrespective of which biomarker was used in the sorting. In other words, rather than each biomarker reporting on different ways to be long‐ or short‐lived, all biomarkers tested appear to correspond to a common gene expression signature of future lifespan.

### 2.3 Biomarker levels correspond with physiological age

We hypothesized that the similarity in gene expression between samples with similar predicted lifespan could be due to a shared state of physiological age. Under the simplest model, all individuals might undergo a similar aging trajectory throughout life, with some proceeding faster than others. Biomarkers of aging would therefore report an individual's position along this trajectory. To the extent that such a common aging trajectory exists, it would necessarily be defined by the average physiological state of a population at any given chronological timepoint. Thus, this model would predict that individuals predicted to be long‐ versus short‐lived at a specific timepoint would be physiologically (transcriptionally, etc.) similar to the average population at a younger or older age, respectively.

**FIGURE 3:**
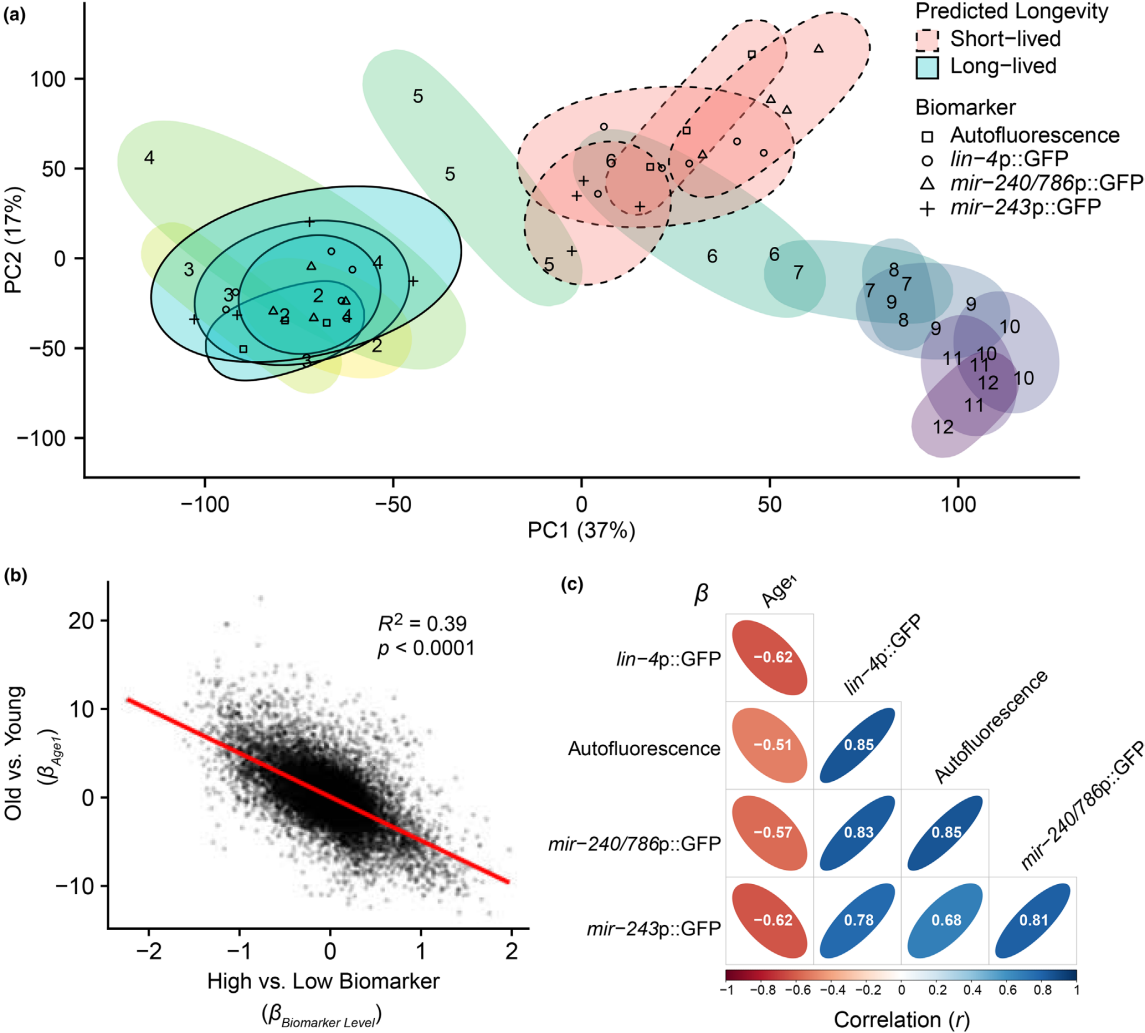
Transcriptomic differences in predicted lifespan correlate to differences in chronological age. (a) PCA plot comparing RNA expression of the average population days 2–12 with sorted animals of different predicted lifespans (chronologically Day 5). Ellipses enclose samples of the same sample group. (b) Scatterplot comparing regression coefficients (*β*) which were used to determine differential expression with respect to chronological age and biomarker level for each gene. These coefficients are analogous to log2 fold‐change. A positive *β*_*Biomarker*_ value indicates a gene more highly expressed in predicted‐long‐lived samples, while a positive *β*_*Age1*_ value indicates increased expression over time. (c) Pairwise comparison *β* coefficient values of high versus low predicted lifespan by each biomarker as well as aging. Intersections are colored according to and labeled with their Pearson correlation coefficient (*r*), indicating positive or negative correlation. *β* coefficient values for long versus short predicted lifespan are correlated across different biomarkers, and the genes more highly‐expressed in the predicted long‐lived subpopulations are the genes that are down‐regulated with aging (and vice versa).

To test this, we compared the gene expression profiles of individuals predicted to be long‐ or short‐lived, all of which were collected at chronological Day 5, with the average population at chronological age days 2–12. PCA revealed that the first principal component separated samples by both age and predicted future lifespan (Figure 3a). This result implies that many of the same transcriptomic features account for differences in both age and predicted lifespan, and that these features make up a plurality of the variance in the data. However, while the predicted long‐lived samples aligned with chronologically younger samples in both PCs 1 and 2, some of the predicted short‐lived samples diverged from the time‐series trajectory in the second principal component, implying some differences between the states of short remaining lifespan and advanced age.

To further explore the relationship between age and predicted lifespan, we used multivariate regression analysis to jointly model the effects of either age or biomarker level on gene expression (Figure S8). We compared the fold‐changes for all detected genes between young and old age with the fold‐changes between high versus low biomarker (here, samples were pooled to compare all predicted‐long‐lived vs. all predicted‐short‐lived samples irrespective of biomarker). We found differential gene expression with respect to aging and with response to biomarker level to be strongly correlated (*R* ^2^ = 0.39), meaning that many of the same genes which vary in expression during aging are also differentially expressed in subpopulations predicted to be long‐ versus short‐lived (Figure 3b). We extended this analysis to each high versus low biomarker pair and found a high degree of similarity in differential gene expression across all biomarkers tested, and similar levels of correlation with age (Figure 3c). Note that the negative, rather than positive, correlation here is the result of the arbitrary assignment of signs to the fold‐change values.

### 2.4 Transcriptional signatures associated with future lifespan

The above analysis indicates that the bulk of the transcriptional signature associated with high versus low biomarker levels is broadly similar to that of aging. We suspect much of these transcriptional changes are relatively “downstream” in the aging process, reflecting the physiological effects of accelerated or retarded aging in the low versus high biomarker populations, rather than the causes of the change in aging rates. In order to identify genetic programs that might be causal, we next attempted to identify systematic differences between high and low biomarker populations that did not simply recapitulate those between young and old individuals. While it is certainly plausible that some of the genes that change with age are also causal in setting the overall rate of aging in the biomarker high versus low populations, distinguishing cause from effect is challenging. In contrast, we reasoned that differences between the biomarker populations in genes that do not change with general aging are unlikely to simply reflect the effects of differential rates of physiological aging, and therefore might be more likely to reflect their underlying causes.

**FIGURE 4:**
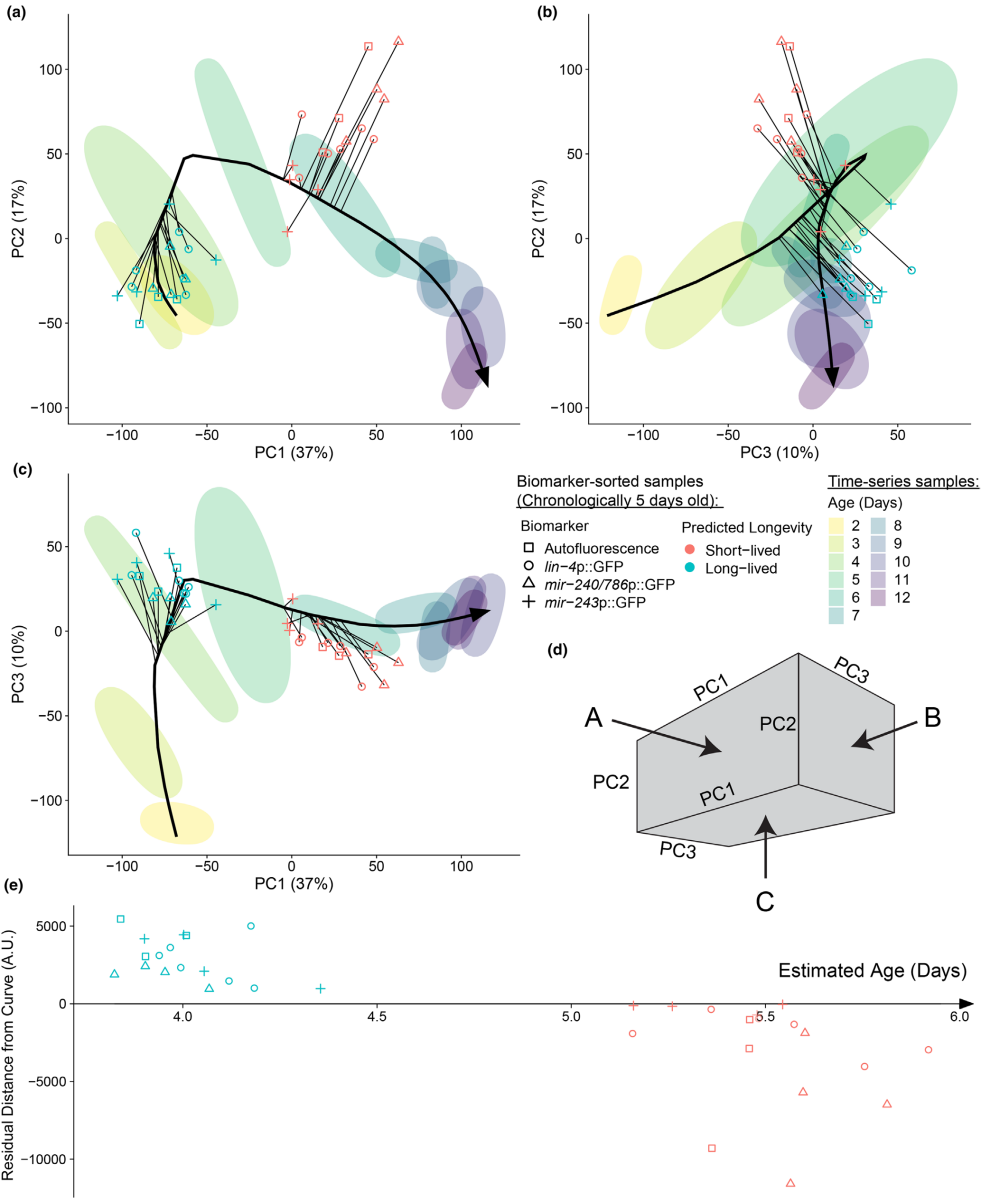
Estimation of physiological age. (a–c) A 3‐dimensional PCA plot viewed from three angles. A principal curve is drawn through the center of the time‐series data, shown here as ellipses. Predicted long‐lived (blue) and short‐lived (red) samples are plotted with a line connecting to the nearest point on the curve, which defines each sample's estimated physiological age. (d) Orientation of the 3‐dimensional plots shown in (a–c). (e) Estimated physiological age (distance along the principal curve) is plotted against distance from the curve for biomarker‐sorted samples (sign of residual corresponds to predicted longevity).

We therefore controlled for the general signature of aging via a two‐step process. First, we determined the apparent physiological age of the biomarker‐high versus–low populations. Next, we compared the biomarker‐high versus low gene‐expression patterns not directly with each other, but with those of unsorted populations at a chronological age matching the physiological age of the sorted biomarker‐high or low populations. To do this, we fit a curve through the center of the time‐series samples in PCA space and used this curve to measure progression along the average aging trajectory (Hastie & Stuetzle, 1989). We then projected all biomarker‐sorted samples onto the nearest location on the curve in order to determine their physiological age (Figure 4 and Figure S1b). We estimated an average physiological age of 4.01 ± 0.14 days across all predicted long‐lived samples and 5.55 ± 0.21 days for the predicted short‐lived samples (Figure 4e).

We performed a second regression analysis, this time regressing gene‐expression against the estimated physiological age of each sample. This identifies genes that distinguish biomarker‐high from–low populations over and beyond what physiological age alone can accomplish (Figures S9 and S1C). If predicted future lifespan as a function of biomarker expression were purely a function of a difference in physiological age, we would expect differential gene expression to be entirely explained by physiological age once it is added as a variable to the model. On the contrary, the differential expression of several genes was still best explained by differences in biomarker level even after controlling for physiological age. Furthermore, we found differential gene expression between samples predicted to be long‐ versus short‐lived was still correlated between all biomarkers tested (compare Figure S9c to Figure S9g). This suggests that the similarity between biomarkers was not just the result of a common association with physiological age, but potentially due to a common transcriptional state correlated with (and perhaps causal of) future differences in lifespan as well.

### 2.5 Enrichment analysis of genes associated with physiological age versus future lifespan

We determined 6162 genes to be significantly differentially expressed (FDR‐adjusted *p*‐value <0.0001) specifically with respect to physiological age (using all three physiological age terms in the regression model), 1407 genes specifically with respect to biomarker level, and 3419 genes in the intersection between the two (Figure S9h). To analyze the gene expression signature of physiological age versus that still associated with biomarker level, we performed gene set enrichment analysis (GSEA) (Subramanian et al., 2005). We ranked the full list of detected genes by their *p*‐values with respect to *β*_Physiological‐age1_—as the linear term in the polynomial equation used to model the effects of age, this coefficient was significant in almost all of the genes associated with physiological age (8571/9581 total) and represents expression either broadly rising or falling with age. We also ranked all genes by their *p*‐values with respect to *β*_Biomarker_ and performed GSEA on both of these lists to assess for GO terms and KEGG pathways significantly enriched in either or both.

**FIGURE 5:**
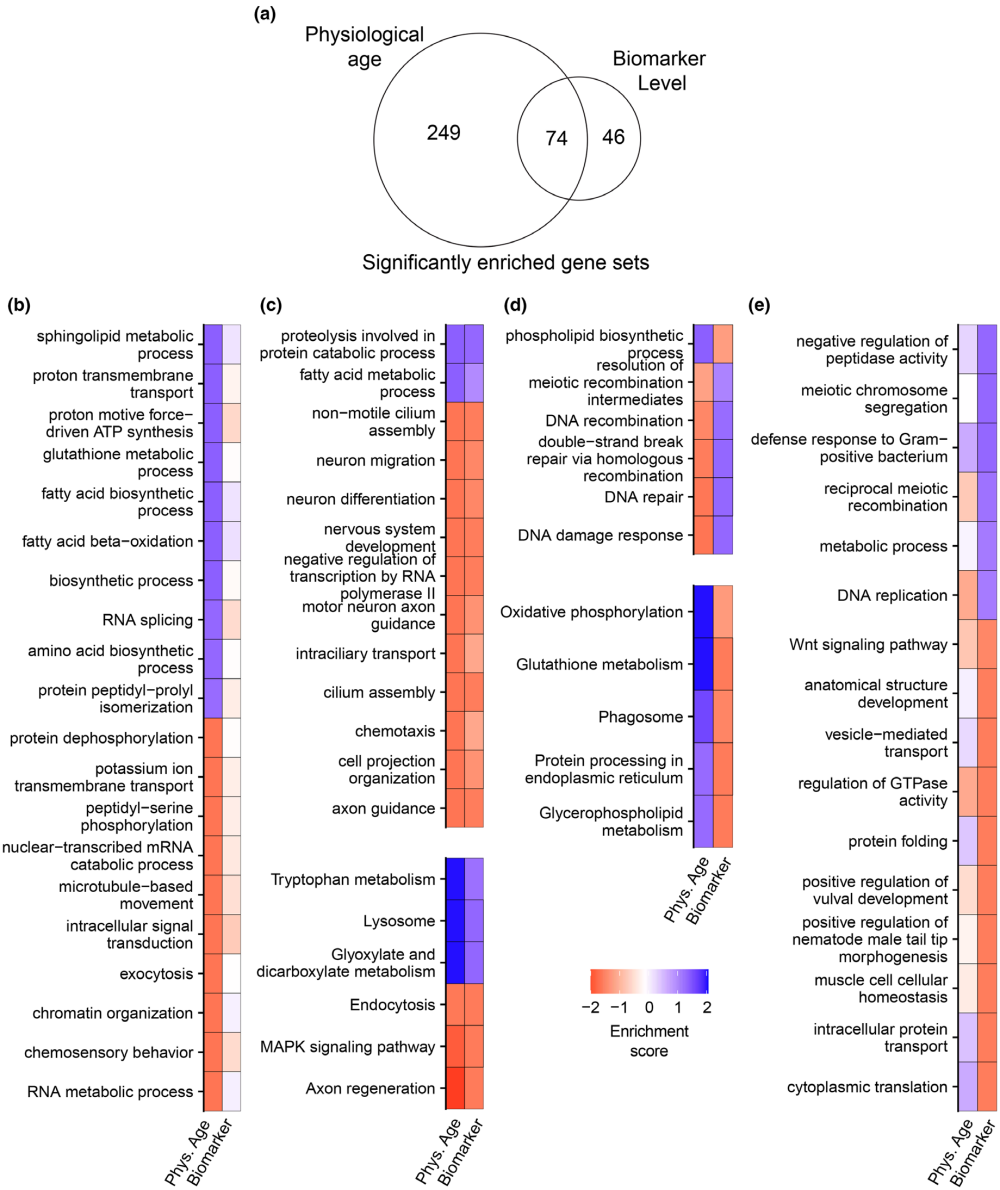
Gene set enrichment analysis (GSEA) of genes associated with physiological age versus biomarker expression. GSEA was performed on the full list of all detected genes ranked by significance of *β*_Physioloical‐age1_ and *β*_Biomarker_. (a) Venn diagram showing the number of GO terms and KEGG pathways associated with physiological age and biomarker level (FDR‐adjusted *p*‐value <0.1). (b–e) Enrichment was calculated as signed −log10 (FDR‐adjusted *p‐*value), such that positive values (blue) represent gene sets enriched in physiologically young worms (declining with age) and/or predicted long‐lived worms, while negative values (red) represent enrichment in physiologically older worms (increasing with age) and/or predicted short‐lived worms. (b) Most significant GO‐biological process (GO:BP) terms associated with physiological age but not biomarker level. (c, d) Most significant GO:BP terms (top) and KEGG pathways (bottom) associated with both physiological age and biomarker level. (c) highlights terms which show enrichment in the same direction; that is, terms which are significantly enriched both in physiologically‐young and predicted‐long‐lived animals or, reciprocally, in physiologically‐old and predicted short‐lived animals. (d) highlights terms which are enriched in opposite directions; in physiologically‐young animals and those predicted to be short‐lived, or in physiologically‐older animals and those predicted to be long‐lived. (e) Most significant GO:BP terms associated specifically with biomarker level but without significant correlation to physiological age.

We found 249 terms associated specifically with physiological age, 46 associated specifically with biomarker level, and 74 in the intersection between the two (Figure 5a), and we listed the most significantly enriched terms in each of these categories (Figure 5b–e). The intersection of this Venn diagram contained two categories of terms: those which trended in the expected direction (Figure 5c) and those which were enriched in opposite directions (Figure 5d). By “expected,” we mean terms that were enriched with increasing physiological age as well as in predicted‐short‐lived animals or vice‐versa—for instance, genes in the MAPK signaling pathway, which regulates various stress responses, are expressed at higher levels in physiologically old animals as well as animals with short remaining lifespan. Less intuitive are gene sets which appeared to trend in opposite directions—terms which were enriched with increased physiological age but also with long predicted lifespan or vice‐versa for physiologically young worms and predicted short lifespan (Figure 5d). Among these were genes annotated for meiotic recombination and DNA damage, both of which appeared to increase with age but were more highly expressed in long‐lived individuals (Figure S10). Finally, there are those gene sets which are differentially expressed specifically with respect to biomarker level but with no apparent association with physiological age (Figure 5e). Many of these genes (751 total, about 15% of the differentially expressed genes with respect to predicted lifespan) showed little to no change over time on average, making them interesting targets for analysis given their correlation with future lifespan across several biomarkers (Figure S11).

To further characterize genes associated with future lifespan, we analyzed differentially expressed genes for the over‐representation of specific stress response pathways. In addition to the aforementioned DNA‐damage response pathway genes, we found that innate immune response pathway and xenobiotic response genes were over‐represented among genes expressed more highly in animals predicted to be long‐lived (Figure S12a–c). Genes expressed at higher levels in short‐lived animals were enriched for genes associated with stress‐activated MAPK response, unfolded protein response, and response to osmotic stress (Figure S12d–f). Using the tissue enrichment analysis tool on WormBase (Angeles‐Albores et al., 2016), we determined that the genes more highly expressed in predicted‐long‐lived animals are typically associated with the muscular system, intestine, and germline (Figure S13). These results were corroborated by comparison to published transcriptomic studies in a variety of perturbations using the online tool WormExp (Yang et al., 2016) (Figure S14). We found high overlap with genes whose expression was altered in response to mutation of immune response genes such as the p38 MAPKK gene *sek‐1* as well as bacterial insult (Wu et al., 2019). We also noted overlap with several datasets which ablated or otherwise perturbed the germline through the mutation of genes such as *glp‐1*, *pgl‐1*, or *mes‐4*.

## 3 DISCUSSION

In contrast to chronological age, which is measured simply by the ticking of a clock, defining physiological age is a much more complex task. To deal with this complexity, we use biomarkers of aging as proxy measurements to determine how physiologically young or old an individual is. However, this requires that we understand what “young” and “old” look like for a given biomarker. Using the average biomarker levels over chronological time to build a trajectory from a youthful to aged state, we can then place an individual on this trajectory and compare whether it is physiologically older or younger than its actual chronological age would suggest. This works because biomarkers of aging vary over time in each individual at a faster or slower rate depending on each individual's rate of aging. Here, we expanded this logic by building a trajectory of aging using the whole transcriptome and comparing the transcriptomes of subpopulations predicted to be long‐ or short‐lived by the expression of four different biomarkers of aging. In doing so, we identified a class of genes which separate along this trajectory of physiological age and another which separates orthogonally to it.

That biomarkers of aging correlate with a common physiological age state is also consistent with results suggesting that various interventions which affect population longevity, such as long‐lived mutants, “rescale” lifespan and healthspan relative to the wildtype population. Stroustrup et al. (2016), for instance, showed that several interventions which lengthen or shorten lifespan rescale the hazard curve of the wildtype population, and more recently Statzer et al. (2022) showed that several long‐lived mutants have proportionally‐scaled healthspan relative to wildtype controls. Tarkhov et al. (2019) performed a meta‐analysis of several RNA‐seq studies of long‐lived mutants and similarly found that the transcriptomic age of these populations was scaled primarily along a single axis in a manner correlated with lifespan extension. Our results suggest a similar phenomenon occurring among untreated individuals of the same population, whereby long‐ and short‐lived individuals undergo, in large part, temporally‐scaled versions of the same transcriptional trajectory. While our interpretations are limited by sorting and sequencing populations only at one time‐point, future work could confirm whether differently‐fated individuals continue to follow this common trajectory by sequencing at later timepoints post‐sorting.

While the finding that a difference in predicted lifespan largely resembles a difference in apparent age may be intuitive, the consistency of this signature across each biomarker tested is notable. One could imagine an alternate model in which each biomarker correlates with a specific age‐related etiology, resulting in several different ways to be healthy or unhealthy—instead, we find the transcriptomic differences underlying high versus low expression of each biomarker tested to be remarkably similar. This result lends further support to previous findings that certain transcriptional biomarkers of aging, even when expressed in different tissues, appear to correlate with some common underlying state related to future lifespan (Kinser et al., 2021).

There have been other reports in the literature of distinct biological states that appear to underlie long versus short life (at least in certain subpopulations). In replicative aging of *S. cerevisiae*, isogenic populations of cells have been shown to age in one of two distinct “modes” characterized by the decline of different organelles and, importantly, different average replicative lifespans (Jin et al., 2019; Li et al., 2020; Zhou et al., 2023). In *C. elegans*, Zhao et al. (2017) identified variability in lifespan and cause of death due to is differential infection by the *E. coli* food source, which the authors hypothesized may be due to differential ability of individuals to resist infection (see also Sánchez‐Blanco & Kim, 2011). While we did not directly test stress tolerance here, Bazopoulou et al. (2019) demonstrated that early‐life redox states generate a hormesis effect whereby oxidized larvae grow into longer‐lived and more stress‐resistant adults than their more reduced siblings. Both this phenomenon and that which we observe in our biomarker‐sorted populations are likely on a spectrum, rather than dichotomous as in the first two examples: while we, like Bazopoulou et al., isolated the tails of the population, most individuals in both studies exhibit intermediate biomarker levels between the extremes.

In addition to the consistent signature of genes that are similarly differentially expressed between both biomarker‐predicted long‐ versus short‐lived subpopulations and chronologically young versus old populations, we also found a distinct set of genes “orthogonal” to the chronological aging axis, that is, genes that distinguish sample groups by biomarker‐predicted lifespan but in a manner unconnected from physiological age. The genes in this set are consistently differentially expressed across all biomarkers tested, which implies that these genes are related to a coherent underlying biological state that enhances or diminishes future lifespan, but which itself does not change over time in the average population. It is tempting to speculate that this set of genes may act to set each individual's rate of aging. Such a trait may well be stable over time in any given individual (and hence not appear to change over time in an aging population) but would of course distinguish long‐ and short‐lived subpopulations.

If these genes are indeed related to rate of aging, it may explain their strong overlap with differentially expressed genes from germline ablation experiments. The reproductive system of *C. elegans* is a well‐studied regulator of aging—ablation of the germline (while leaving the somatic gonad intact) extends lifespan, and the onset of reproduction has been linked with a reduction of somatic proteostatic capacity and a conversion of intestinal biomass into yolk (Ezcurra et al., 2018; Hsin & Kenyon, 1999; Labbadia & Morimoto, 2015). It has even recently been argued that *C. elegans* has a semelparous life history, characterized by a single bout of reproduction followed by a programmed “reproductive death” for the benefit of its offspring (Kern et al., 2023). The germline has also been previously linked to heterogeneity in the aging process, both in terms of variable reproductive capacity (Pickett et al., 2013) and also variability in the appearance of chromatin masses in the uteri of aged worms (Golden et al., 2007). This latter phenomenon could be linked to the overrepresentation of genes associated with DNA damage repair and meiotic recombination which we observed in worms predicted to be long‐lived. Moreover, Eder et al. (2024) recently demonstrated through single‐worm single‐cell transcriptomics that decoupling of gene expression between the germline and soma is a major driver of nongenetic interindividual heterogeneity in *C. elegans* aging and that ablation of the germline is sufficient to blunt variability in lifespan. Our finding of a set of genes related to the germline which varies with future lifespan “orthogonal” to the axis of physiological age fits perfectly with a model in which a decoupling of somatic and germline transcription drives variability in aging.

It is important to note certain caveats given the transcriptomic nature of this work: one important limitation is that we only measured mRNA levels, which do not necessarily correlate to the activity of the proteins for which they encode. Relatedly, gene set enrichment analysis is limited by the accuracy and completeness of category annotations, and increased detection of transcripts annotated for a given pathway or process is not sufficient evidence to claim increased pathway activity. Further experimentation is required to better understand what role if any these germline‐related genes play in the determination of individual lifespan. One approach would be to perform longitudinal analysis of biomarker expression in germline‐less and/or feminized hermaphrodites. This germline‐centric hypothesis predicts that, absent germline‐mediated signaling, the biomarkers we have analyzed should no longer predict future lifespan.

## 4 MATERIALS AND METHODS

### 4.1 *C. elegance* strains and maintenance

All strains used were maintained at 20°C on NGM plates seeded with *E. coli* OP50 using standard culture techniques (Brenner, 1974). VT1474 (*mir‐243*p::GFP), VL370 (*mir‐240/786*p::GFP), and BA671 (*spe‐9*(*hc88*)) were obtained from the Caenorhabditis Genetics Center (CGC), and VT1474 and VL370 were originally created by Martinez et al. (2008). Worms carrying *zaIs1* (*lin‐4*p::GFP) were obtained from and originally generated by the Slack lab (Boehm & Slack, 2005).

All strains assayed were crossed with BA671, which contains a temperature sensitive allele of *spe‐9* (hc88) to prevent self‐fertilization at the restrictive temperature of 25°C (Fabian & Johnson, 1994). Individual culture and longitudinal imaging of animals in the “worm corral” was carried out as described previously (Kinser et al., 2021; Pittman et al., 2017).

### 4.2 Microfluidic sorting

Worms were synchronized by hypochlorite treatment of gravid adults, and L1 larvae were allowed to hatch and arrest overnight in S‐Basal medium. L1 larvae were plated on 10 cm NGM plates at a density of 1500 worms per plate and cultured at 25°C. At 5 days post‐replating, plates were washed with M9 buffer containing 0.02% TWEEN 20 detergent (Sigma), and collected worms were allowed to settle by gravity and washed with M9 buffer to remove bacteria and particulate matter. Worms were placed in a syringe pump and fed through a single‐layer microfluidic device mounted on a Leica DMi8 inverted microscope with a 5× objective (Figure S6). The microfluidic chip was made using PDMS elastomer cast on a mold of SU‐82050 photoresist fabricated using standard soft lithography techniques (Jenkins, 2013). Sorting was handled automatically using code written in Python. Images were captured using a Zyla‐5.5 sCMOS camera (Andor), and GFP and red autofluorescence (TRITC) images were visualized using the standard DMi8 filter cube (Leica). Fluorescence intensity was measured as the 95th percentile brightest pixel in the worm body, the area of which was determined by subtraction of the background image taken when no worm was present.

The first 100 worms for any assay were used to calibrate “high” and “low” biomarker expression, which was defined as the top and bottom tenth percentile of the distribution, respectively. Measurements for subsequent worms were added into the distribution, meaning that the thresholds for “high” and “low” change slightly over the course of the sort. The microfluidic device has three output channels—individuals with “high” or “low” biomarker expression were sorted into the left or right channels and recovered, while all others were sorted through the middle output channel and discarded (Figure S6).

### 4.3 Survival assays

Animals were recovered from the microfluidic sorter in 50 mL conical tubes and replated onto NGM + 50 μg/mL kanamycin plates seeded with kanamycin resistant OP50‐NeoR *E. coli* obtained from the CGC. These conditions were chosen in order to circumvent the risk of bacterial contamination in the sorting equipment. Immediately after replating, animals were assessed for movement to ensure they survived the sorting procedure and otherwise censored. Sorted populations were maintained at 25°C, and all animals were assayed daily for motion and scored dead if they failed to respond to gentle stimulus. Animals were censored if they escaped or burst. Statistical differences in survival curves were determined by log‐rank test using the “SciPy” package for Python (Virtanen et al., 2020).

### 4.4 RNA‐seq

Immediately after sorting, recovered “high” and “low” biomarker populations were resuspended in 100 μL of M9 buffer to which 400 μL of TRIzol reagent (ThermoFisher) was added. The resultant mixture was repeatedly flash‐frozen in liquid nitrogen, thawed at 37°C, and vortexed vigorously for at least five freeze–thaw cycles to lyse the worms. Total RNA was obtained by phenol chloroform extraction and purified using the RNeasy Micro kit (Qiagen). For time‐course samples, animals were synchronized by hypochlorite treatment and grown on 10 cm NGM plates. Every 24 h post‐replating, plates were washed with M9 buffer, and RNA was extracted as described above. RNA quality was assessed using a 4200 TapeStation system (Agilent), and library construction and sequencing was carried out by Novogene.

### 4.5 Analysis of gene expression data

Genes were considered expressed if 75% of samples had at least five RNA‐seq counts and were excluded otherwise, resulting in 14,085 “detected” genes. Differential expression analysis was carried out using the R package “DESeq2” (Love et al., 2014). For each gene, expression (RNA‐seq counts) was modeled as a function of age (days 2–12 post‐hatch, encoded as a continuous variable), biomarker level (1 or −1 for predicted long‐lived or short‐lived samples, respectively, or 0 for time‐course samples), and a categorical variable which accounted for sequencing batch. To account for non‐linear changes in gene expression with time, the effect of age on gene expression was modeled as a third‐degree polynomial. Thus, the following model was fit:

$$\begin{array}{c} Y_{\exp}=x_{\mathrm{Age}}^{3}\beta_{\mathrm{Age}3}+x_{\mathrm{Age}}^{2}\beta_{\mathrm{Age}2}+x_{\mathrm{Age}}\beta_{\mathrm{Age}1}\\ +x_{\text{Marker}}\beta_{\text{Marker}}+x_{\text{Batch}}\beta_{\text{Batch}}+\beta_{\text{Intercept}} \end{array}$$

*β* coefficients were then estimated and significance determined by Wald test to determine the effects of aging, biomarker level, and batch on the expression of each gene. To determine the similarity between different biomarkers, a similar model was fit with *X*_Marker_ broken into a series of dummy variables such that *β* coefficients for each high‐low biomarker pair were determined separately:

$$\begin{array}{c} Y_{\exp}=x_{\mathrm{Age}}^{3}\beta_{\mathrm{Age}3}+x_{\mathrm{Age}}^{2}\beta_{\mathrm{Age}2}+x_{\mathrm{Age}}\beta_{\mathrm{Age}1}+x_{\mathrm{lin}-4}\beta_{\mathrm{lin}-4}+x_{\mathrm{mir}-243}\beta_{\mathrm{mir}-243}\\ +x_{\mathrm{mir}-240/786}\beta_{\mathrm{mir}-240/786}+x_{\text{Autofluorescence}}\beta_{\text{Autofluorescence}}+x_{\text{Batch}}\beta_{\text{Batch}}+\beta_{\text{intercept}} \end{array}$$

Normalized counts were obtained using the variance stabilizing transformation (“vst”) function from DESeq2, and batch correction was performed using the “removeBatchEffect” function from the R package “Limma” (Law et al., 2018).

For clustering analysis, normalized counts for time‐series samples were averaged at each timepoint for each gene. A smoothing spline with 5 degrees of freedom was fit to the average trend for each gene, and the resulting splines were standardized to have a mean of 0 and standard deviation of 1. Genes were automatically placed into cluster 0 (no correlation with time) if none of their *β*_*Age*_ coefficients had an associated FDR‐adjusted *p*‐value <0.01, which totaled 2511 genes. The remaining 11,574 genes were clustered using the “tsclust” function of the R package “dtwclust” (Sarda‐Espinosa, 2024). Distance was calculated using dynamic time warping with a window size of 1 day, and hierarchical clustering was performed using complete linkage, using DTW barycenter averaging to calculate cluster centroids. After clustering was complete, the tree was cut into five clusters.

### 4.6 Estimation of apparent age

Principal component analysis was carried out for all samples, and a curve was fit through the center of the time‐series (unsorted) data using the R package “princurve” (Cannoodt & Bengtsson, 2019; Hastie & Stuetzle, 1989). All samples were then projected to the nearest point on the curve, and physiological age was estimated proportional to distance along the arc. To separate the effects of physiological age and predicted lifespan on gene expression, the same regression analysis as above was carried out, with the exception that these estimated physiological age values were used in place of chronological age for each sample.

### 4.7 Enrichment analysis

Gene set enrichment analysis (GSEA) was carried out for full lists of genes ranked by their FDR‐adjusted *p*‐values associated with *β*_*Age1*_ and *β*_*Marker*_ using the online tool EasyGSEA (https://tau.cmmt.ubc.ca/eVITTA/, Cheng et al., 2021). Specifically, GSEA was carried out with respect to standard gene ontology terms for biological processes, molecular function, and cellular compartment, as well as for KEGG pathway enrichment. To assess for overrepresentation of specific stress pathways, we used the hypergeometric test to compare the list of differentially expressed genes with respect to biomarker level (*β*_*Marker*_ FDR‐adjusted *p*‐value <0.0001) to the genes annotated for a variety of stress response pathways using GO:BP terms (Figure S11g). Tissue enrichment analysis was carried out using the enrichment analysis tool available on WormBase (https://wormbase.org/tools/enrichment/tea/tea.cgi, Angeles‐Albores et al., 2016). The same lists of genes were assessed for overlap with published transcriptomic data using the online tool WormExp (https://wormexp.zoologie.uni‐kiel.de/wormexp/, Yang et al., 2016).

## AUTHOR CONTRIBUTIONS

Matthew C. Mosley: Conceptualization, formal analysis, validation, investigation, visualization, methodology, writing—original draft, writing—review and editing, experimental design, data collection, writing of analytical software. Holly E. Kinser: investigation, visualization, methodology, writing—review and editing, data collection, data curation. Olivier M.F. Martin: formal analysis, methodology, visualization, writing—review and editing, writing of analytical software. Nicholas Stroustrup: writing—review and editing. Tim Schedl: writing—review and editing, supervision. Kerry Kornfeld: writing—review and editing, supervision, project administration. Zachary Pincus: conceptualization, supervision, funding acquisition, methodology, writing—original draft, writing—review and editing, project administration, experimental design, writing of analytical software, image acquisition and analysis software.

## FUNDING INFORMATION

This work was supported by NIH grant R01 AG057748 and a Beckman Young Investigator award from the Arnold and Mabel Beckman Foundation.

## CONFLICT OF INTEREST STATEMENT

The authors declare no conflict of interest.

## Supporting information


Appendix S1.

Appendix S2.

Appendix S3.

## Data Availability

All data and code are available upon request. RNA‐seq count data has been deposited at the Gene Expression Omnibus (GEO) with accession numbers GSE254501 and GSE283419.
