# Supplementary material for: Similarities and differences in the gene expression signatures of physiological age versus future lifespan
Source: Aging Cell. 2024 Dec 6;24(4):e14428. doi: 10.1111/acel.14428 (PMC11984696; doi:10.1111/acel.14428)
Supplement: Supplementary file 3 — Appendix S3. [file ACEL-24-e14428-s002.docx]

| Biomarker | Replicate | Level^a^ | N^b^ | Censored^c^ | Mean  Lifespan^d^ | Standard  Deviation^d^ | Median  Lifespan^d^ | % Difference^e^ | Maximum  Lifespan^d^ |
| --- | --- | --- | --- | --- | --- | --- | --- | --- | --- |
| *lin-4*p::GFP | 1 | High | 74 | 0 | 12.84 | 1.84 | 13 | 30^***^ | 17 |
|  |  | Low | 97 | 2 | 10.58 | 2.22 | 10 |  | 17 |
|  | 2 | High | 108 | 5 | 12.14 | 3.29 | 12 | 50^***^ | 22 |
|  |  | Low | 115 | 1 | 8.46 | 1.74 | 8 |  | 17 |
| *mir-243*p::GFP | 1 | High | 67 | 6 | 13.89 | 2.65 | 14 | 55^***^ | 18 |
|  |  | Low | 70 | 4 | 9.98 | 3.24 | 9 |  | 20 |
|  | 2 | High | 75 | 2 | 11.16 | 2.25 | 12 | 50^***^ | 16 |
|  |  | Low | 90 | 3 | 9.15 | 1.84 | 8 |  | 14 |
| *mir-240/786*p::GFP | 1 | High | 115 | 2 | 9.71 | 3.44 | 8 | 137^***^ | 21 |
|  |  | Low | 56 | 1 | 18.65 | 3.28 | 19 |  | 25 |
|  | 2 | High | 100 | 1 | 9.69 | 2.9 | 9 | 89^***^ | 20 |
|  |  | Low | 125 | 2 | 16.03 | 3.79 | 17 |  | 22 |
| Autofluorescence | 1 | High | 121 | 0 | 9.46 | 1.31 | 10 | 20^***^ | 13 |
|  |  | Low | 85 | 0 | 11.89 | 2.48 | 12 |  | 19 |
|  | 2 | High | 111 | 2 | 10.05 | 3.17 | 9 | 67^***^ | 22 |
|  |  | Low | 68 | 3 | 14.69 | 3.72 | 15 |  | 22 |

Supplemental Table 1. Lifespan statistics for populations sorted by biomarker fluorescence.

1. “High” and “low” refer to the top or bottom 10^th^ percentile of fluorescence relative to the rest of the population
2. Total number of animals in each sample group
3. Number of right-censored animals
4. All lifespans reported in days post-synchronization as L1 larvae
5. Percent difference in median lifespan. *** indicates *p* < 0.0001 as determined by log-rank test.


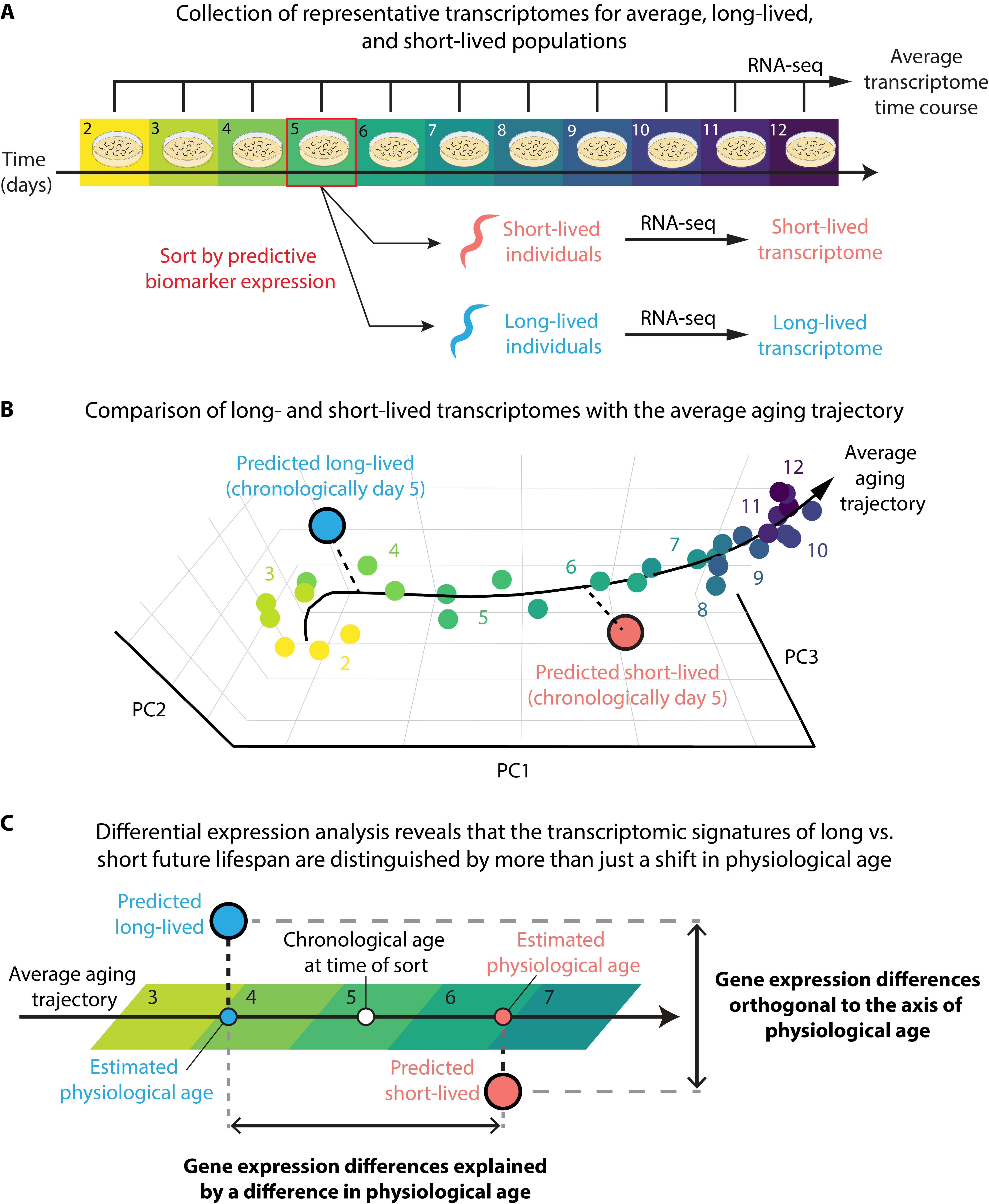


Supplemental Figure 1. Experimental design and analysis pipeline. A) We sampled the whole population at days 2-12. We know there exists the aforementioned variation in each of these samples, and thus we assume a “regression to the mean” transcriptional age as mRNA levels from individuals of varying future lifespans are pooled together. We also separated individuals of the same chronological age (5 days old) into predicted long-lived and short-lived sub-populations using four different fluorescent biomarkers of aging. B) We used PCA to summarize the gene expression profiles associated with chronological ages days 2-12. Using this as the “average aging trajectory,” we then compared where long- and short-lived populations fell along this curve in order to assign a physiological age to each sample. C) We modeled gene expression as a function of physiological age and biomarker expression (predicted future lifespan). We found that, while correlated with future lifespan, a difference in physiological age alone was not sufficient to explain all differential gene expression between samples predicted to be long- vs. short-lived. We thus categorized differentially expressed genes between long- and short-lived samples into two classes: the first contains those genes whose expression reflects a difference in physiological age. The second, on the other hand, contains genes whose expression distinguished long- and short-lived samples in a manner uncorrelated with a difference in physiological age; that is, “orthogonal” to the physiological aging trajectory.


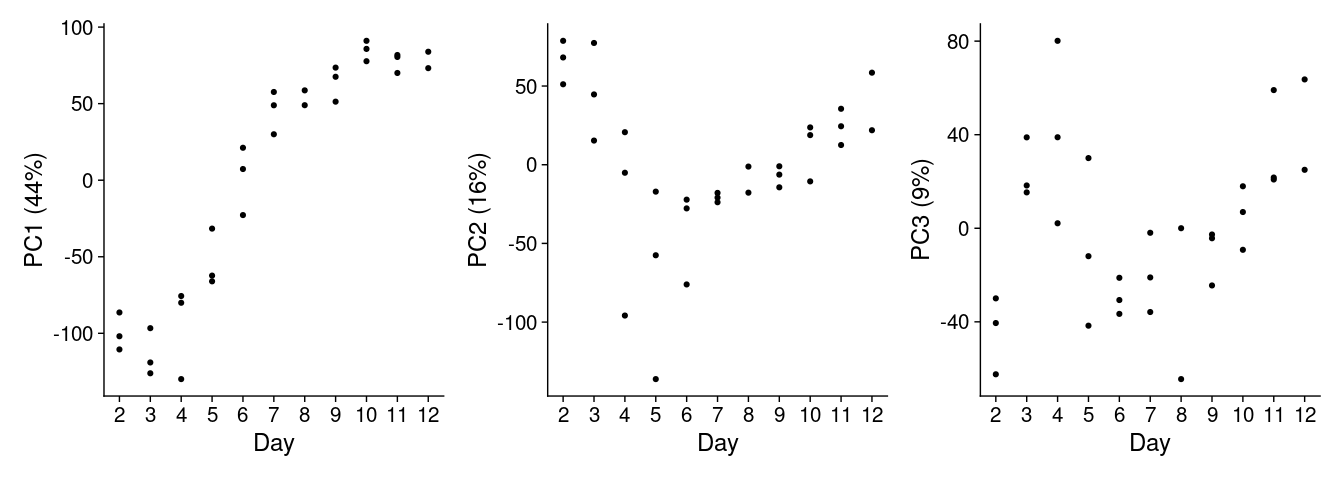


Supplemental Figure 2. Trajectory of average population transcriptional profiles through time. Principal component analysis was carried out on populations days 2-12. Scores for the first, second, and third principal component are plotted over time. Y-axis labels also indicate percentage of variance explained in the data by each principal component.


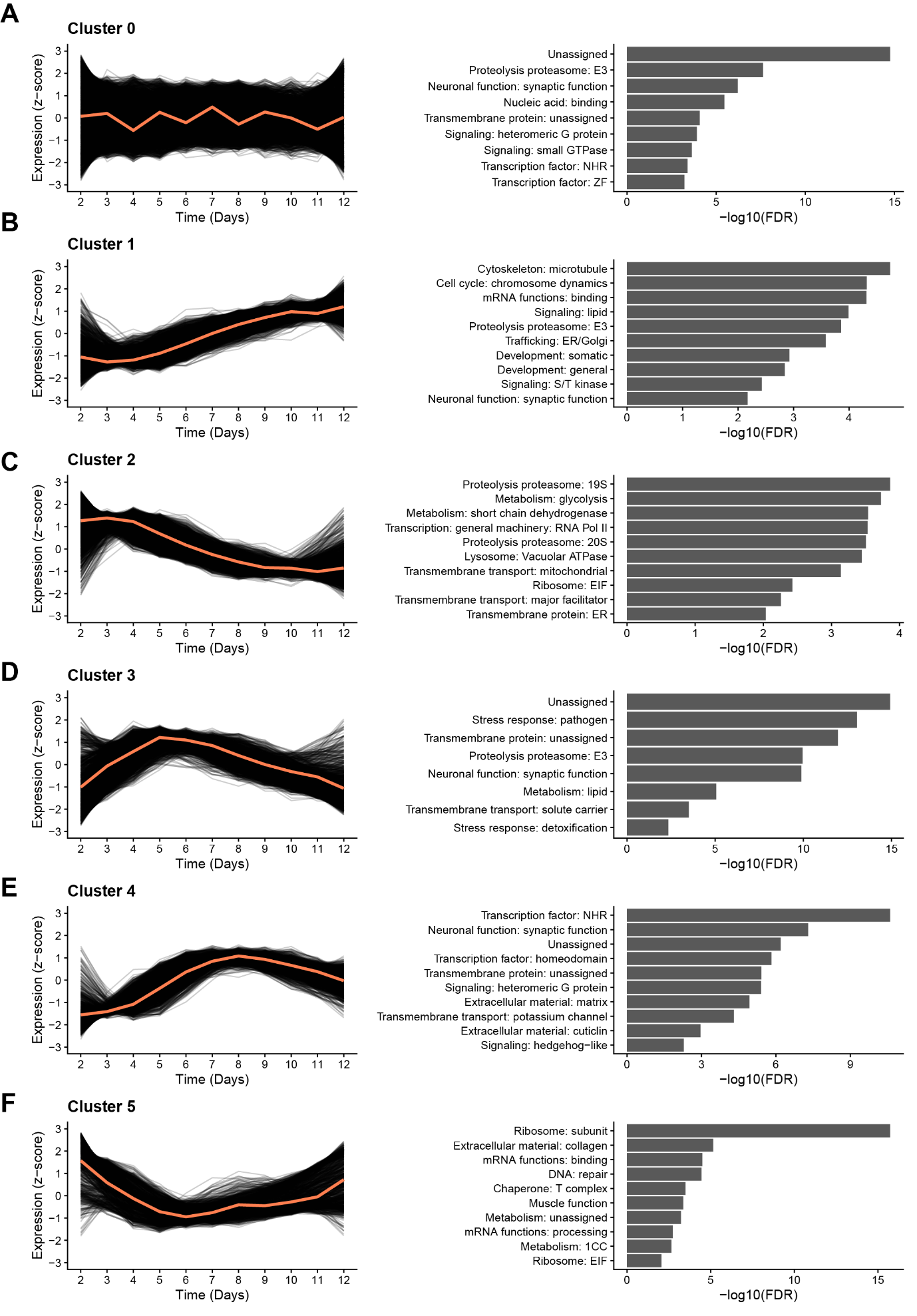


Supplemental Figure 3. Enrichment analysis of temporal gene expression clusters. All genes assigned to each cluster were analyzed using WormCat (Holdorf et al., 2020) to determine functional enrichments, with the top ten terms for each cluster plotted here.


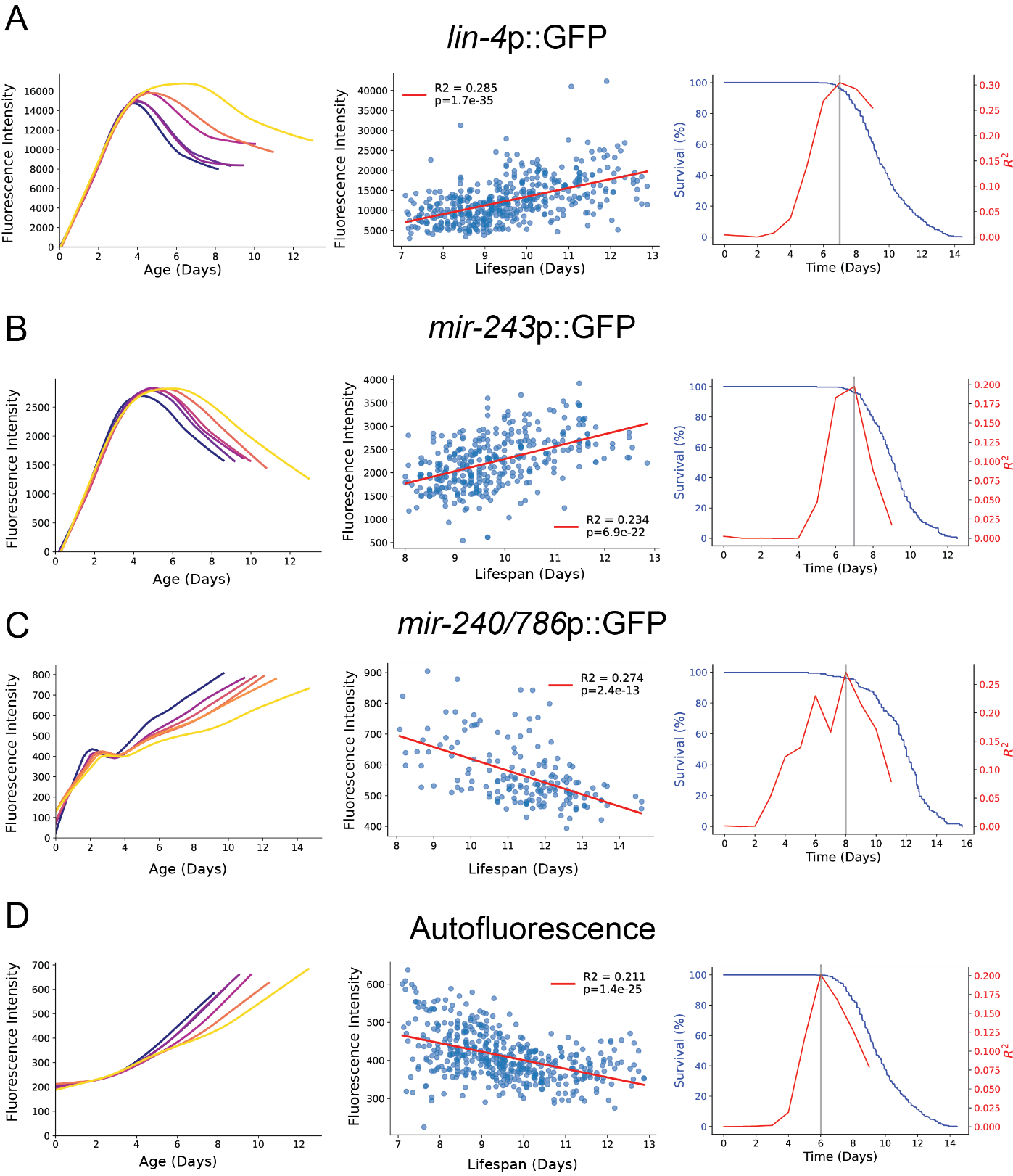


Supplemental Figure 4. Selection of predictive biomarkers of aging. A-D) Summary data for four biomarkers of aging characterized by individual culture in the “worm corral” system. Left column: Animals were divided into six evenly-sized bins by lifespan. Mean biomarker fluorescence intensity (see methods) is plotted over time since birth for each bin, with color warmth corresponding to lifespan. Each line in A and D is the average of 81 animals, lines in B are the average of 64 animals, and lines in C average 30 animals (Rows A and D represent fluorescence measurements made on the same set of experiments). Middle column: Biomarker fluorescence is plotted against eventual lifespan for each animal, with linear regression plotted in red. Fluorescence intensity is an average of fluorescence measurements taken over the course of 24 hours. Right column: Survival curves (blue) are plotted alongside *R^2^* statistics (red) for the correlation between biomarker fluorescence and future lifespan at each timepoint. The vertical grey line corresponds to the timepoint used in the middle column.


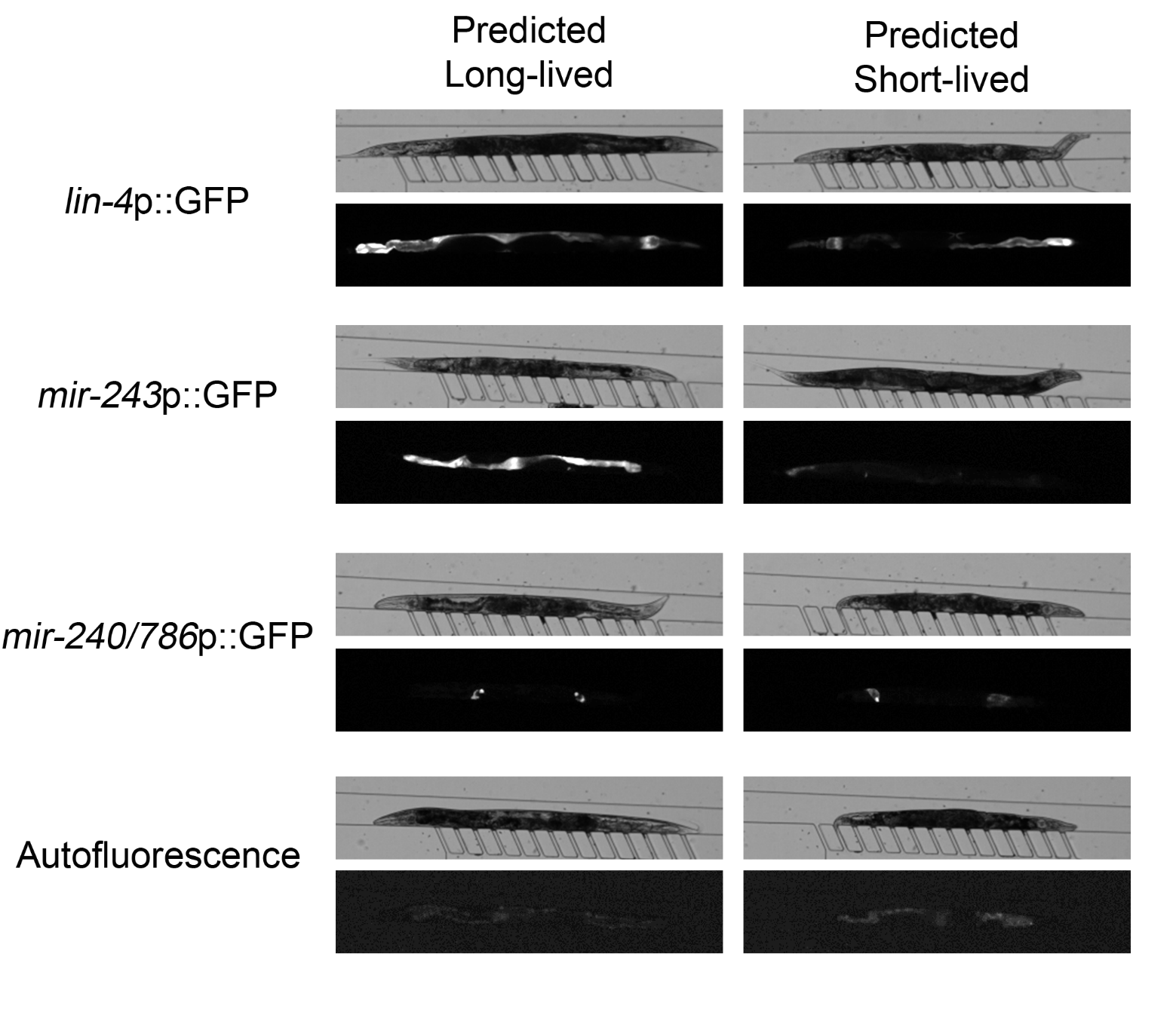


Supplemental Figure 5. Representative images of biomarker fluorescence. Images were taken automatically during sorting. Each row shows a brightfield image and corresponding fluorescent image, cyan (470nm) for GFP and green/yellow (550nm) for autofluorescence. Animals were predicted to be long- or short-lived if they fell above the 90^th^ or below the 10^th^ percentile of fluorescence intensity relative to the rest of the population.


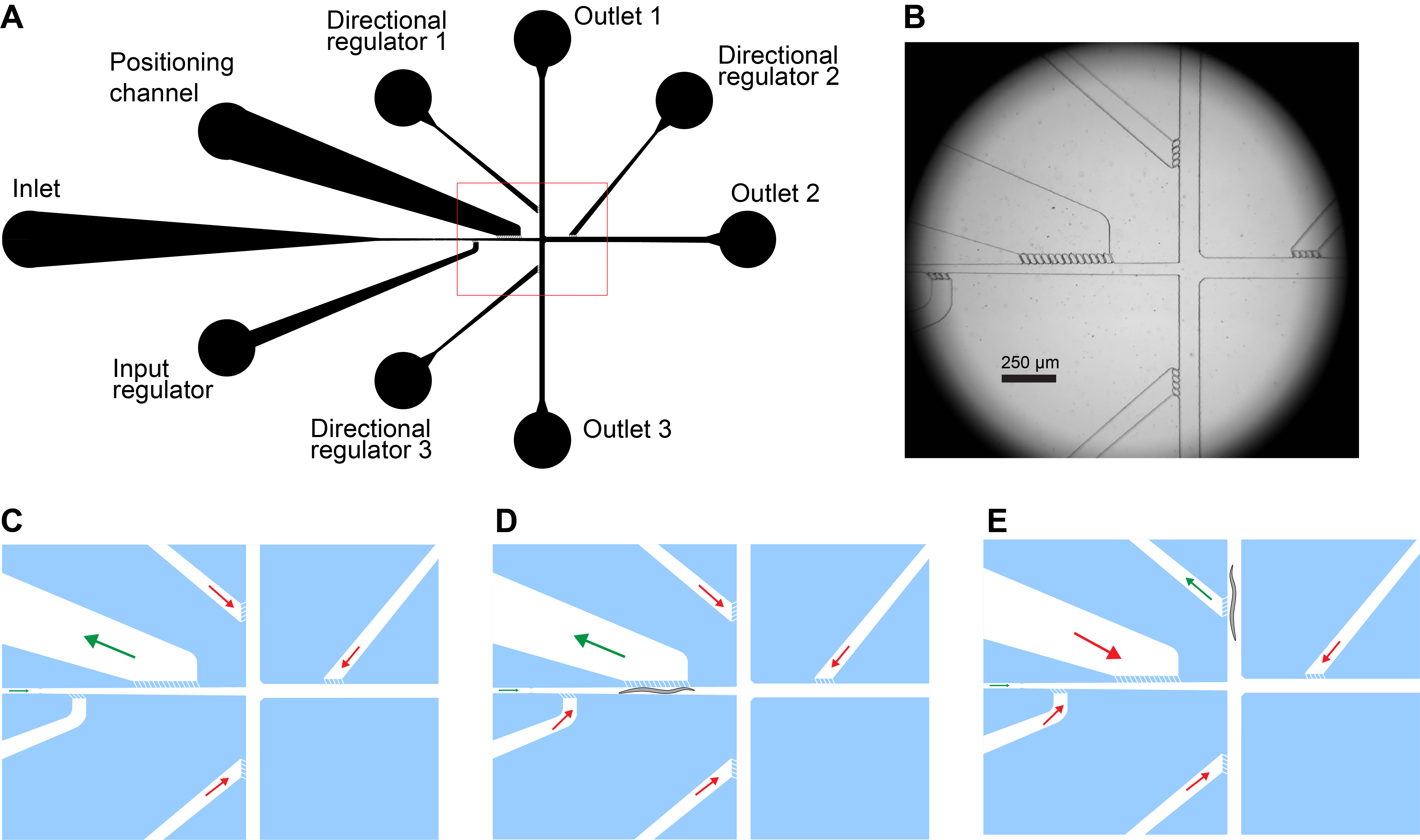


Supplemental Figure 6. Microfluidic sorter design. (A) Full design of the single-layer microfluidic system, which is a PDMS chip on a glass slide. The chip has one input and three outputs, and flow is controlled by differential pressure from channels covered by grating which prevents worms from passing through. Continuous pressure is supplied to a syringe containing a suspension of worms, which is connected to the inlet. The positioning channel and directional regulators 1-3 are connected to valves which can either supply pressure or suction to limit or direct the net flow of fluid. The input regulator channel, meanwhile, is connected to a valve which either supplies pressure to stop the input flow or is in a neutral state. All regulatory channels are connected to a pressure control system coordinated by a program written in Python. (B) The imaging window as seen by the inverted microscope, where animals are positioned, analyzed, and sorted. (C-E) Explanation of the pressure-controlled sorting process. (C) Depicts the system when waiting for a worm to enter the imaging window. Suction is applied at the positioning channel, and all directional channels have a positive output pressure to prevent worms passing this point. (D) When an animal enters the imaging window (detected by the sorting program as a significant change from the background image), the input regulator channel is supplied with pressure to prevent the flow of additional worms. Images are taken in brightfield and fluorescence channels while the worm is immobilized against the grating. (E) The animal is sorted into one of three output channels by switching the positioning channel to positive pressure and one of the directional channels to suction (in this example, directional regulator 1, sorting the worm upwards). When the system returns to the “waiting” state (C), the worm is carried through the selected outlet into a reservoir for collection.


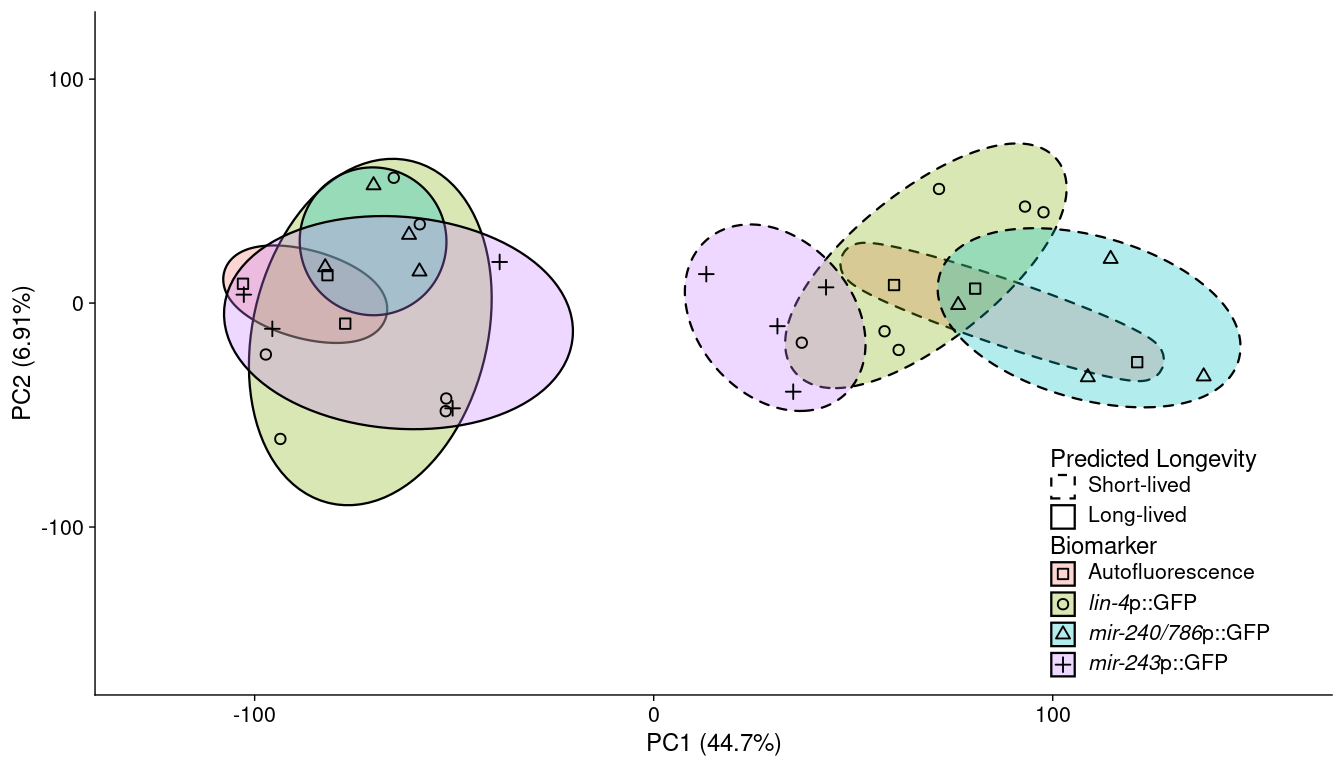


Supplemental Figure 7. Principal component analysis on the transcriptomes of subpopulations predicted to be long- vs. short-lived after sorting by biomarker expression at day 5 post-synchronization. Axes are labeled with percent of variance captured by the first two principal components, with the first component appearing to separate samples by predicted lifespan. Ellipses were drawn to encircle samples with biomarker and predicted lifespan in common.


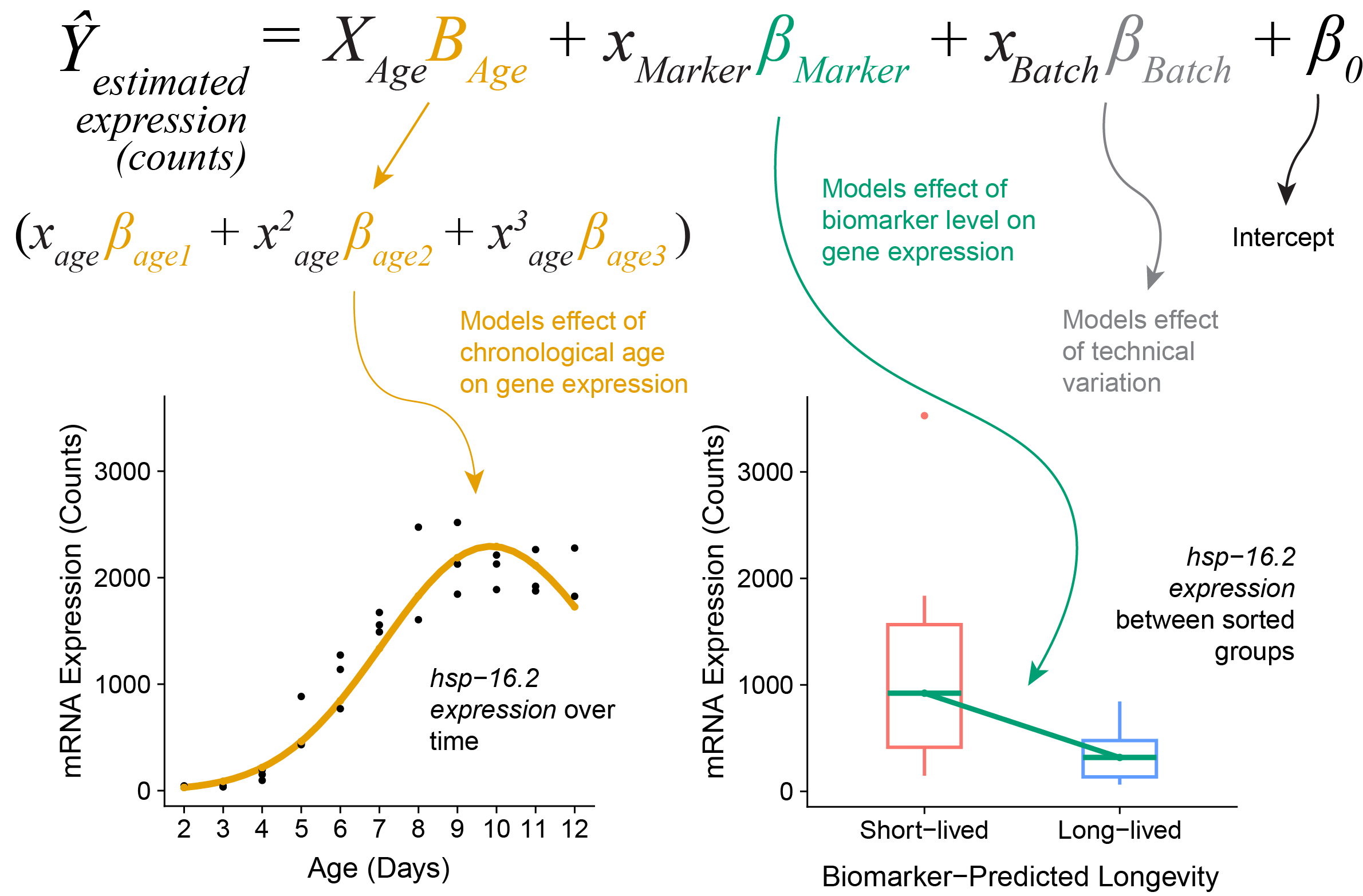


Supplemental Figure 8. Explanation of the regression model used to determine differential gene expression. We used the R package DESeq2 to model gene expression based on a combination of factors; specifically, chronological age, biomarker expression level, and sequencing batch. In the equation above, we already know the *x* values for each sample, as well as each sample’s actual observed gene expression counts (plotted in the two charts). The aim of regression is to solve for the *β* coefficients which result in the best approximation of gene expression (here called *Ŷ)* given the *x* variables. In other words, *β* coefficients are values which explain the relationship between a set of independent variables and a dependent variable. In the context of differential gene expression, *β* coefficients are analogous to fold-changes; they allow us to determine the impact of each variable on gene expression. Importantly, by comparing the *β* coefficients associated with different variables, we can also determine how correlated the two effects are. Because gene expression over time is often nonlinear, we modelled the effect of age on gene expression as a cubic polynomial; thus, age has three *β* coefficients associated with it. To model the effects of biomarker expression/predicted lifespan on gene expression, we encoded the *x*_marker­_ variable as -1 for short-lived samples, 1 for long-lived samples, and 0 for non-sorted (time-series) samples. Finally, the batch variable allows us to control for technical variation between sequencing runs. The *x_marker_ β_marker_* term could also be replaced with terms for the individual biomarkers (*x_lin-4_ β_lin-4_ + x_mir-243_β_mir-243_,* etc.) in order to compare the similarity of long vs short future lifespan as predicted by each marker.


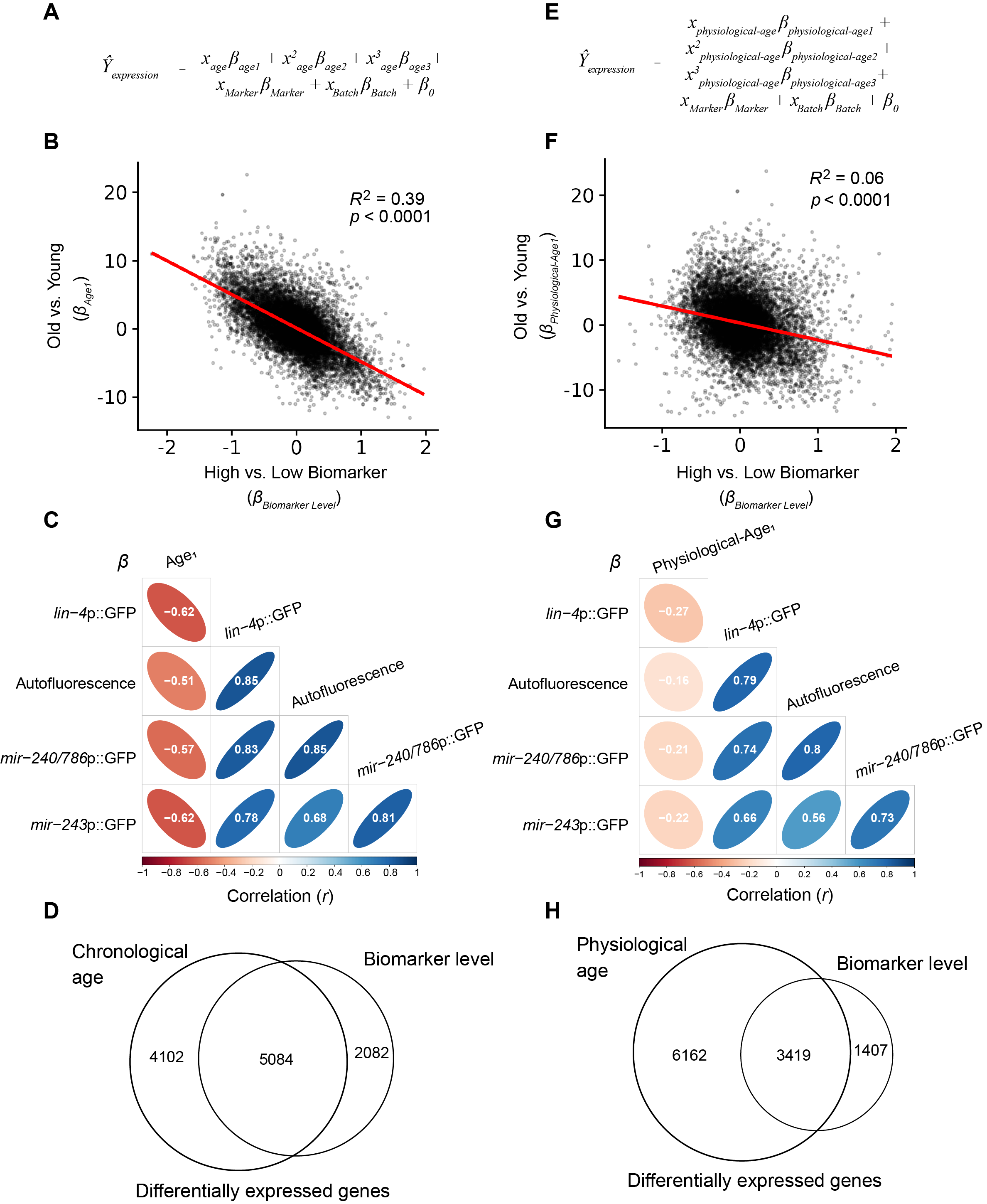


Supplemental Figure 9. Differential gene expression before (A-D) and after (E-H) accounting for physiological age of sorted samples. A) Regression model comparing the effects of chronological age with biomarker expression. B) Regression showing non-independence *β* coefficients in the model. C) Similar regression to (B), extended to each biomarker individually—each high/low pair is highly correlated with one another and also highly correlated with the signature of chronological age (here measured by *β_Age1_*). D) Number of differentially expressed genes associated with chronological age (all *β* coefficients) and biomarker level (FDR-adjusted p-value < 0.0001). E) New regression model accounting for the effects of physiological age after assigning each sample a physiological age value based on similarity to the time-series. F) The *β_Age1_* coefficient, now accounting for physiological age, no longer correlates with *β_Biomarker_*. G) *β* coefficients for each individual biomarker have lost their correlation with *β_Physiological-Age1_*; however, they retain their correlation with each other. H) Differentially expressed genes associated with physiological age (all *β* coefficients) vs biomarker level.


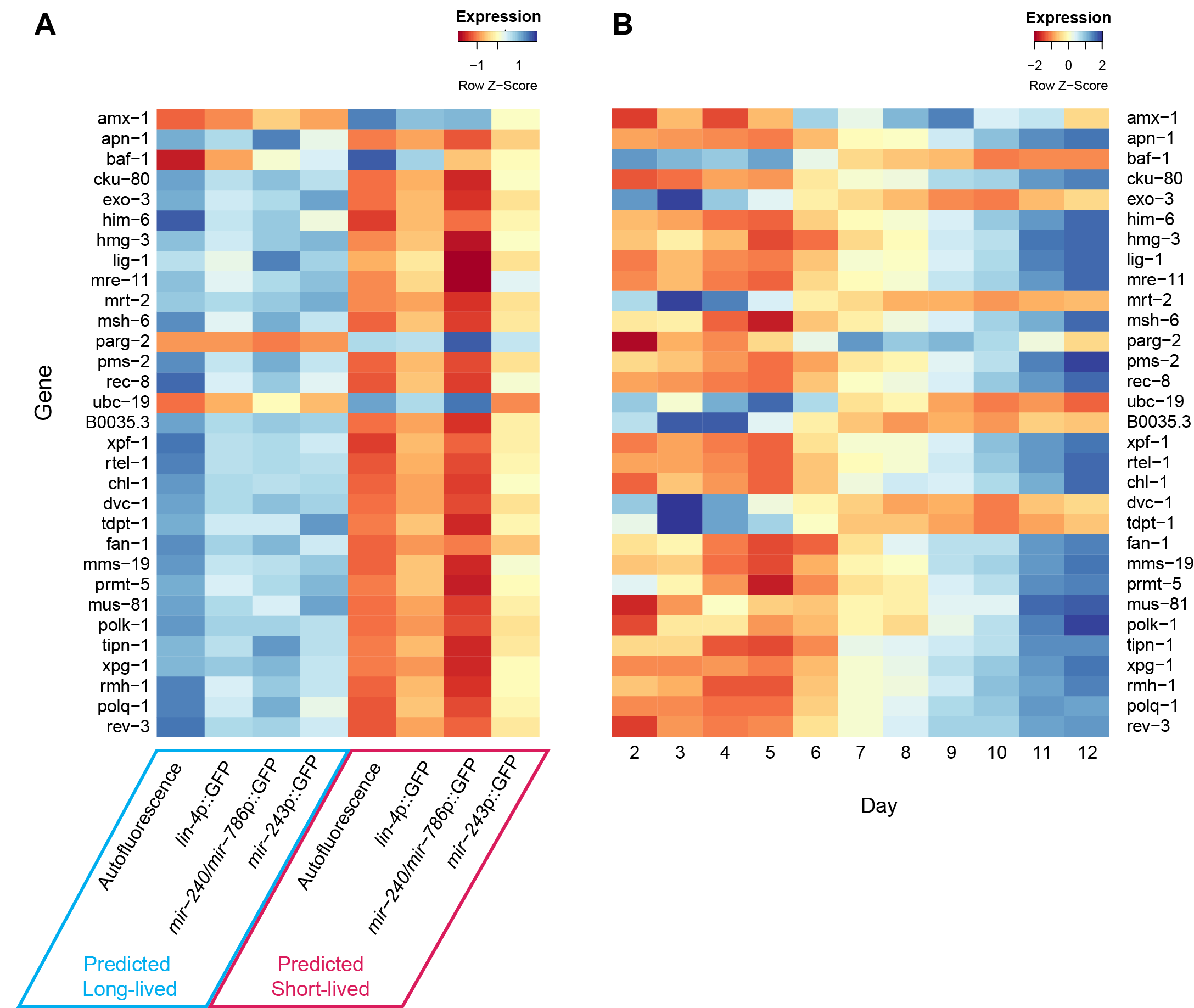


Supplemental Figure 10. DNA damage response genes are expressed more highly in long-lived sub-populations but associated with older physiological age. Heatmap of genes annotated with the GO-biological process term “DNA damage response” which were significantly differentially expressed with respect to biomarker level (A) and physiological age (B). Each cell represents the average of samples in that group, and values are normalized within each row relative to their respective heatmap.


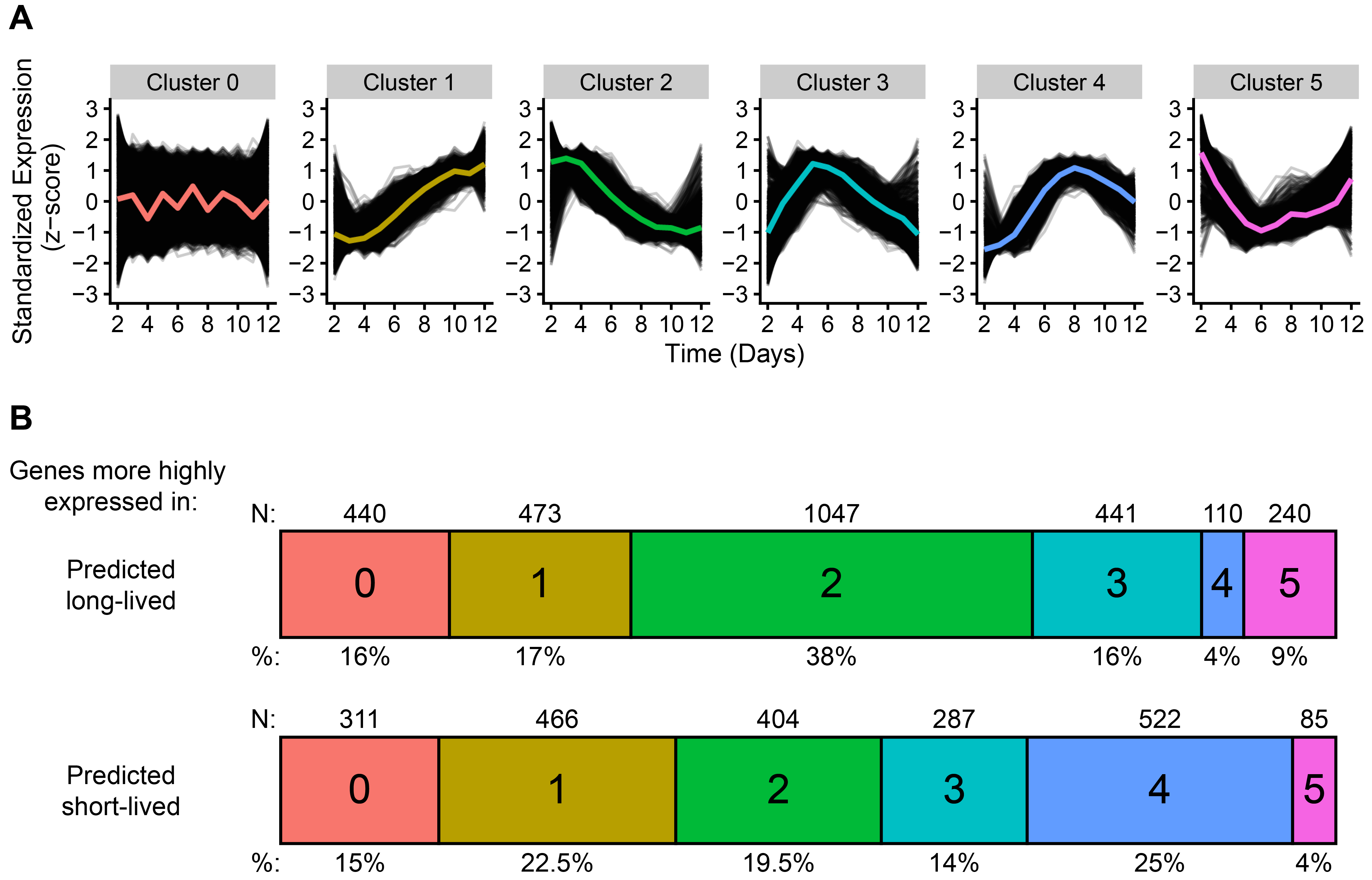


Supplemental Figure 11. Time-series cluster membership of genes associated with long vs. short future lifespan. A) Expression of each gene over time was classified into one of six clusters. B) Number of differentially expressed genes with respect to biomarker level (FDR-adjusted p-value < 0.0001) in each cluster.


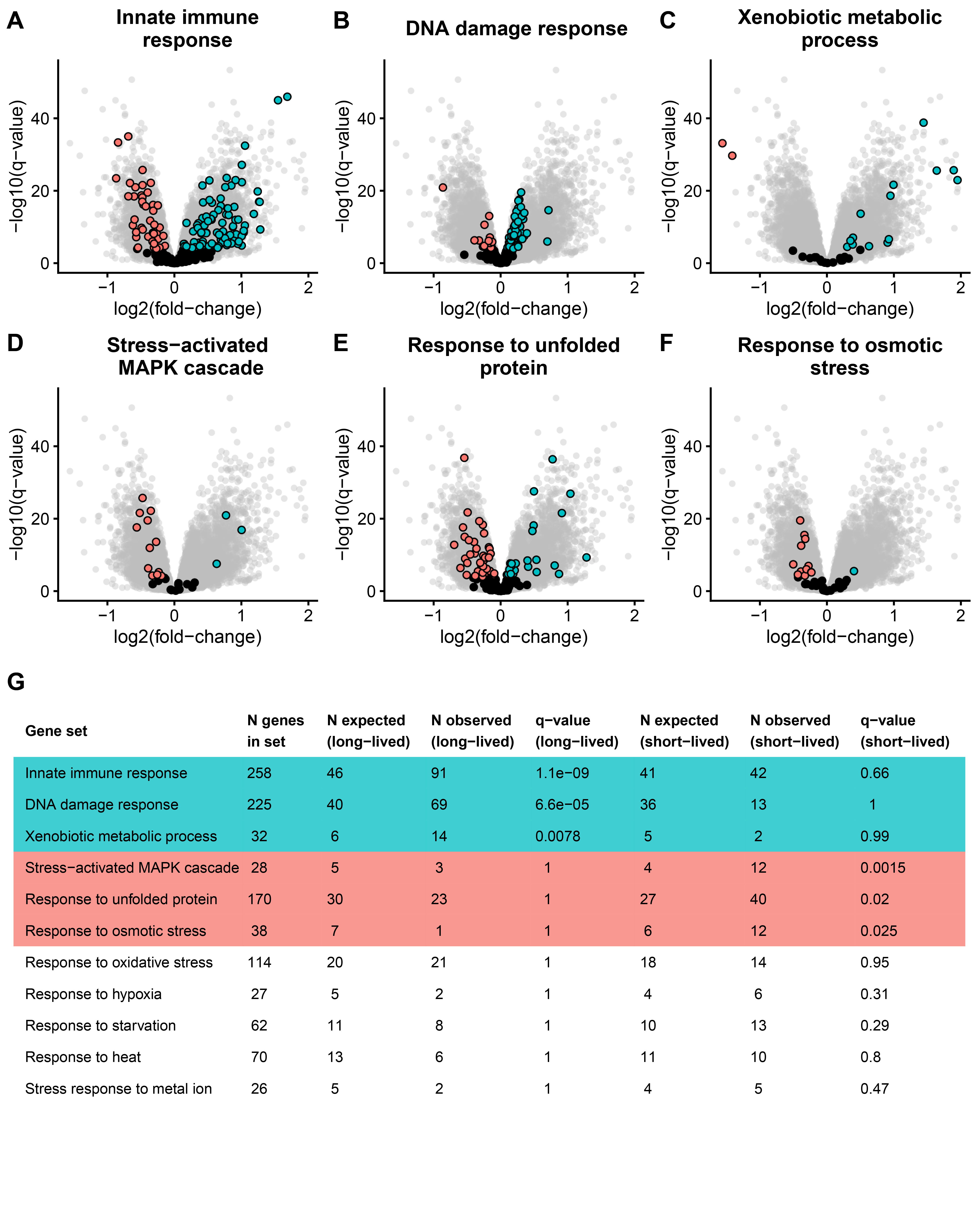


Supplemental Figure 12. Enrichment of stress response pathways in genes correlated with future lifespan. (A-F) Volcano plots of gene expression fold-changes relative to biomarker level, with positive and negative log2(fold-change) values indicating higher or lower expression in animals predicted to be long-lived, respectively. In each plot, genes with an annotation for the indicated gene ontology term are highlighted in blue or red if they were determined to be significantly differentially expressed (q < 0.0001) in the positive or negative direction, respectively. Genes in the set but below this threshold are colored in black, and detected genes not in the given set are in grey. (G) Table of stress pathways tested for over-representation. The lists of 2751 genes found to be more highly expressed in predicted long-lived animals and 2075 genes found to be more highly expressed in short-lived animals were assessed for overlap with each gene set using the hypergeometric test. “N genes in set” refers to all detected genes annotated for the indicated pathway, “N expected” is the number of genes one would expect to detect by random chance given the number of candidate genes (by hypergeometric distribution), whereas “N observed” is the actual number of DEGs with that annotation. The q-value is the result of the hypergeometric test, indicating the (FDR-adjusted) probability of observing an overlap of this size or greater by random chance. Terms with a q-value < 0.05 are highlighted in blue and red depending on if the significantly overlapping list was positively or negatively associated with future lifespan, respectively.


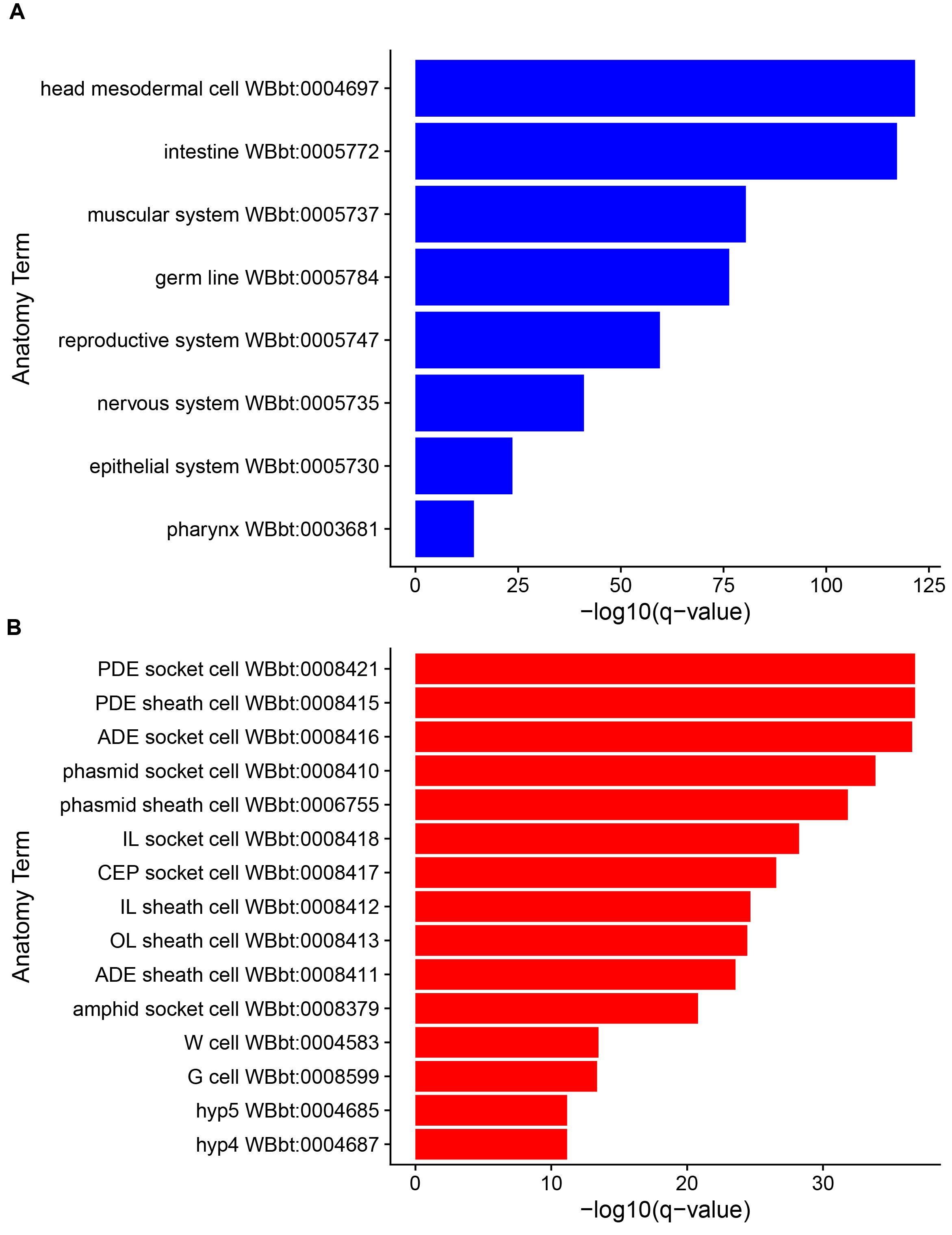


Supplemental Figure 13. Tissue enrichment analysis. Differentially expressed genes with respect to biomarker level (FDR-adjusted p-value < 0.0001) were analyzed for association with specific tissues and/or cell types using the tissue enrichment analysis tool at wormbase.org (Angeles-Albores et al., 2016). A) Anatomy term enrichment in genes expressed more highly in predicted long-lived animals. B) Enrichment from genes expressed more highly in short-lived animals.


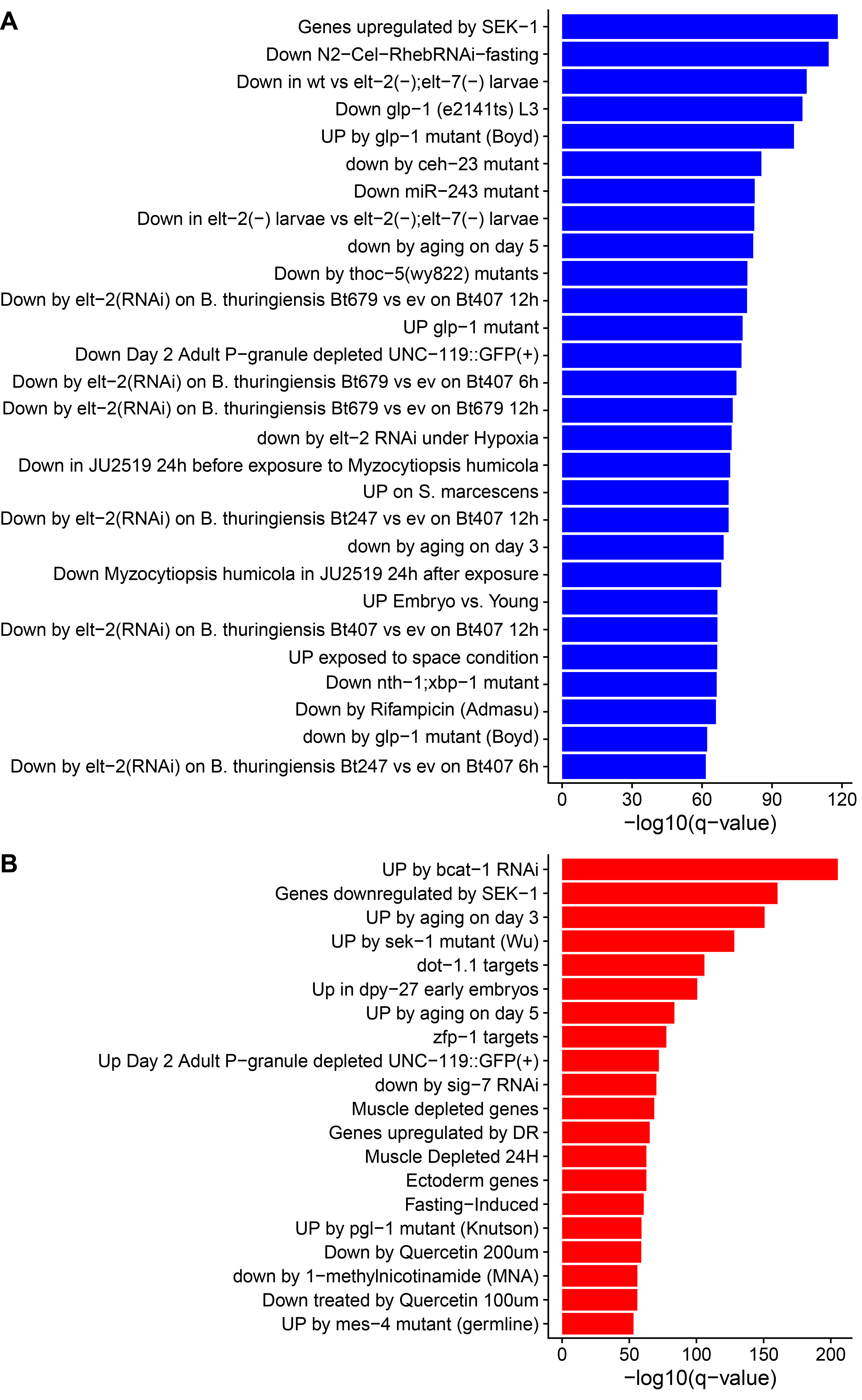


Supplemental Figure 14. Comparison with published datasets. Differentially expressed genes with respect to biomarker level (FDR-adjusted p-value < 0.0001) were analyzed for overlap published transcriptional datasets using WormExp (Yang et al., 2016). A) Genes upregulated in predicted-long-lived and B) predicted-short-lived animals were compared to published transcriptional datasets using a hypergeometric test.
